# Irradiation Selects for p53-Deficient Hematopoietic Progenitors

**DOI:** 10.1371/journal.pbio.1000324

**Published:** 2010-03-02

**Authors:** Andriy Marusyk, Christopher C. Porter, Vadym Zaberezhnyy, James DeGregori

**Affiliations:** 1 Department of Biochemistry and Molecular Genetics, University of Colorado Denver School of Medicine, Aurora, Colorado, United States of America; 2 Program in Molecular Biology, University of Colorado Denver School of Medicine, Aurora, Colorado, United States of America; 3 Department of Pediatrics, University of Colorado Denver School of Medicine, Aurora, Colorado, United States of America; 4 Integrated Department of Immunology, University of Colorado Denver School of Medicine, Aurora, Colorado, United States of America; Fred Hutchinson Cancer Research Center, United States of America

## Abstract

Identification and characterization of mutations that drive cancer evolution constitute a major focus of cancer research. Consequently, dominant paradigms attribute the tumorigenic effects of carcinogens in general and ionizing radiation in particular to their direct mutagenic action on genetic loci encoding oncogenes and tumor suppressor genes. However, the effects of irradiation are not limited to genetic loci that encode oncogenes and tumor suppressors, as irradiation induces a multitude of other changes both in the cells and their microenvironment which could potentially affect the selective effects of some oncogenic mutations. P53 is a key tumor suppressor, the loss of which can provide resistance to multiple genotoxic stimuli, including irradiation. Given that *p53* null animals develop T-cell lymphomas with high penetrance and that irradiation dramatically accelerates lymphoma development in *p53* heterozygous mice, we hypothesized that increased selection for p53-deficient cells contributes to the causal link between irradiation and induction of lymphoid malignancies. We sought to determine whether ionizing irradiation selects for *p53-*deficient hematopoietic progenitors in vivo using mouse models. We found that p53 disruption does not provide a clear selective advantage within an unstressed hematopoietic system or in previously irradiated BM allowed to recover from irradiation. In contrast, upon irradiation p53 disruption confers a dramatic selective advantage, leading to long-term expansion of *p53-*deficient clones and to increased lymphoma development. Selection for cells with disrupted p53 appears to be attributable to several factors: protection from acute irradiation-induced ablation of progenitor cells, prevention of irradiation-induced loss of clonogenic capacity for stem and progenitor cells, improved long-term maintenance of progenitor cell fitness, and the disabling/elimination of competing p53 wild-type progenitors. These studies indicate that the carcinogenic effect of ionizing irradiation can in part be explained by increased selection for cells with p53 disruption, which protects progenitor cells both from immediate elimination and from long-term reductions in fitness following irradiation.

## Abstract 2

While disruption of p53 is selectively neutral within non-stressed hematopoiesis, it confers a strong selective advantage upon irradiation, leading to expansion of p53 mutant clones and lymphoma development.

## Author Summary

Cancer progression can be understood through the framework of Darwinian evolution, which involves two major factors: genetic mutation and selection. Random mutations are thought to result in the initiation and phenotypic diversification of tumors, and environmental influences mediate selection for those mutations that increase tumor cell fitness. Since oncogenic mutations are necessary for the development of spontaneous malignancies and since experimental introduction of these mutations often leads to transformation and cancers, the causation of cancers by carcinogens is traditionally attributed to their induction of new mutations that are oncogenic. We instead asked whether selection for oncogenic mutations is affected by ionizing irradiation, an archetypal mutagenic carcinogen, by examining the selective effects of inactivation of the critical tumor suppressor gene *p53*. While disruption of p53 is selectively neutral in populations of unstressed hematopoietic progenitors, it provides a strong selective advantage upon irradiation. This selection of *p53*-deficient clones is attributable to protection from irradiation-induced cell death and loss of cellular fitness. Importantly, the selective expansion of irradiated cells bearing p53 disruption is blocked in the presence of non-irradiated wild-type competitors, indicating that the disabling of competing wild-type cells by irradiation is critical for selection of p53-deficient cells. Our results argue that induction of cancers by irradiation involves selection for mutations that confer radioresistance, and suggest that greater focus on how carcinogenic contexts impact on selection is warranted in understanding, preventing and treating cancers.

## Introduction

Exposure to ionizing radiation (including γ or X rays) is strongly associated with cancer etiology in humans and mouse models [1],[2]. Since cancer development requires the accumulation of oncogenic mutations and mutagen exposure has been shown to cause cancer, predominant paradigms attribute the carcinogenic action of mutagenic carcinogens (including radiation) to the induction of genetic and epigenetic alterations in oncogenes and tumor suppressor genes [1],[3],[4]. On the other hand, various investigators have proposed that carcinogenic treatments increase the selective advantages conferred by certain oncogenic mutations, thereby initiating tumorigenesis [5]–[9]. While ionizing irradiation is an archetypal mutagenic carcinogen, the causal link between induction of mutations in oncogenic loci and carcinogenesis is mostly inferential. On the other hand, ionizing irradiation clearly induces multiple changes both within cells and in their microenvironment [1],[10]. Thus, the carcinogenic effect of irradiation might not be limited to causation of mutations in cancer-related genes but may also be attributed to increased selection for certain oncogenic events, which are either preexisting or irradiation-induced.

P53 is a critically important tumor suppressor that mediates responses to a variety of cellular stresses and has well-characterized roles in mediating cell cycle arrest and apoptosis in response to genotoxic stress [11]. The *p53* gene is mutated in about half of human tumors, and many tumors that retain wild-type (WT) *p53* contain mutations that disrupt p53 regulation. A number of studies have documented that loss of *p53* function confers a survival advantage following γ-irradiation in short-term survival assays [11]. In particular, *p53* confers a dramatic protection of thymocytes from γ-irradiation induced apoptosis in vivo [12]–[14]. Ex vivo, *p53* null hematopoietic cells are resistant to irradiation-induced death and to loss of clonogenic potential [14]–[18]. On the other hand, short-term resistance to genotoxic stress conferred by *p53* mutation often does not correlate with long-term survival advantages [19], which might reflect the frequent incompatibility of extensive DNA damage with long-term survival.

Germline disruption of p53 in mice leads to lethal thymomas and sarcomas with 100% penetrance [20]–[22]. While γ-irradiation accelerates development of malignancies in newborn *p53*−/− mice, this acceleration is not seen in adult *p53*−*/*− mice [23]. However, irradiation dramatically accelerates tumorigenesis in *p53* heterozygous (+/−) adult mice, and most of the resulting tumors exhibit loss of the second *p53* allele [23], suggesting that loss of p53 function may be selected for following irradiation. Alternatively, the acceleration of thymoma development in *p53*+/− mice by irradiation might be explained by the promotion of loss-of-heterozygosity at the *p53* locus or by the induction of oncogenic mutations, in either case due to the mutagenic effects of irradiation. This latter possibility is supported by the observation that many oncogenic mutations that normally activate apoptotic or senescence responses can drive strong proliferation in cells with disrupted p53 function [24].

The relative importance for induction versus selection of oncogenic mutations in the carcinogenic action of irradiation remains poorly explored. In particular, whether the causal link between radiation exposure, p53 disruption, and cancers involves selection for p53 loss or depends entirely upon irradiation-induced mutagenesis at loci encoding proliferation control genes remains unresolved. To address this question, we analyzed the impact of irradiation on the selective effect of p53 disruption in a minor fraction of hematopoietic progenitor cells within predominantly WT hematopoietic pools. This approach models the physiological context whereby malignancies are initiated by rare cells with oncogenic mutations. Our experiments demonstrate that following irradiation, p53 loss provides an immediate and sustained selective advantage in all hematopoietic lineages, which translates into greater expansion of *p53-*deficient clones and increased lymphoma development.

## Results

### Irradiation Selects for p53 Disruption in Hematopoietic Cells

**Figure 1:**
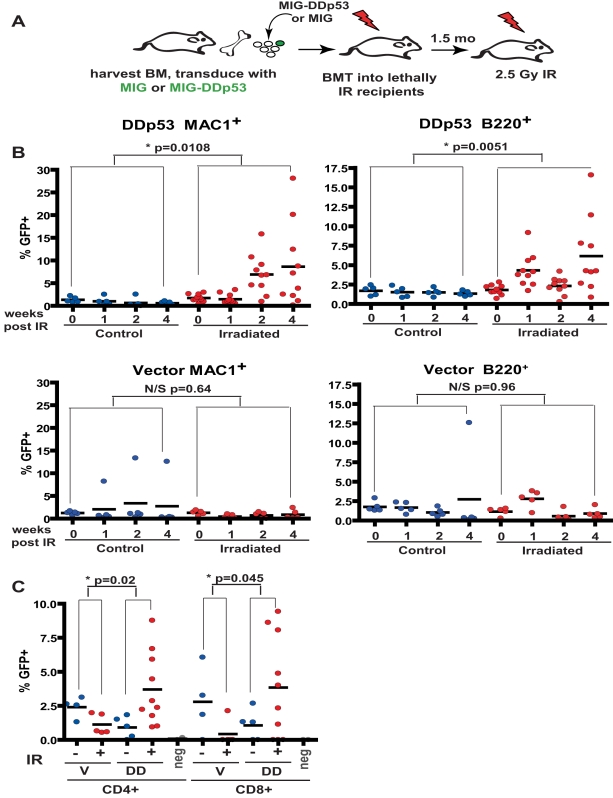
X-irradiation selects for hematopoietic progenitors expressing DDp53. Freshly isolated BM was transduced with MiG (Vector) or MiG-DDp53 and transplanted into lethally irradiated recipients (schemata in A). Initial transduction efficiency was 3.8% for the vector and 3.2% for the DDp53 transduced cells. 1×10^7^ cells were transplanted per recipient. Six weeks post-transplantation, blood was drawn for the baseline analysis (0 wk). Subsequently, five recipients of vector transduced BM and 10 recipients of DDp53 transduced BM were X-irradiated with a single 2.5 Gy dose, and five vector and five DDp53 mice were left untreated. (B) At 1, 2, and 4 wk post-irradiation, peripheral blood was analyzed for the expression of GFP in MAC^+^ myeloid and B220^+^ B-lineage cells. (C) At 55 d post-irradiation, GFP expression in peripheral blood CD4^+^ and CD8^+^ T cells was analyzed (a later time point is analyzed, as production of significant mature T cells requires at least 6 wk post-irradiation). The “neg” data points are from mice that received no transplantation, which serve as negative controls for GFP detection. For B, the indicated *p* values are for *t* tests comparing means of changes in GFP percentages (baseline to 4 wk post-irradiation) between irradiated and control groups. For C, *p* values are for the comparison of the changes in GFP percentages upon irradiation between vector and DDp53 groups.

To address the impact of irradiation on selection of cells with dysfunctional p53, we generated mice with mosaic hematopoietic systems, containing a small percentage of cells with disrupted p53 activity and co-expressed GFP (Figure 1A). To do so, we transplanted bone marrow (BM) progenitors transduced with low titer MSCV-ires-GFP retroviruses (MiG) encoding DDp53 (or empty vector controls) into lethally irradiated recipients. DDp53 encodes for the multimerization domain of p53 (amino acids 302-390), and expression of DDp53 leads to potent inhibition of endogenous p53 activity [25],[26]. The transplanted animals were allowed to recover for 6 wk, at which point hematopoiesis was restored with relatively normal peripheral leukocyte counts (unpublished data). At this point, roughly 2% of the cells were GFP^+^ both in myeloid and B-cell lineages (Figure 1B, time 0). Thus, this model creates a context wherein the fate of a small percentage of p53 disrupted hematopoietic progenitors can be monitored in an otherwise WT background and that also eliminates potential effects of p53 deficiency in non-hematopoietic tissues [27],[28].

The mice were then sub-lethally X-irradiated (2.5 Gy), and the percentage of transduced (GFP^+^) cells was monitored over 4 wk in peripheral blood cells. The percentages of DDp53 transduced cells (GFP^+^) in the non-irradiated controls remained stable (Figure 1B; control groups), indicating that p53 disruption does not provide a substantial advantage during normal steady state hematopoiesis. In contrast, irradiation led to a significant increase in the percentages of DDp53 cells, as average percentages of GFP^+^ cells increased 5-fold in the myeloid (Mac1^+^ cells) lineage and 3.5-fold in the B cell (B220^+^ cells) lineage (Figure 1B; irradiated groups). Examples of flow cytometric profiles are shown in . Given that irradiation had no effect on the expansion of vector transduced cells, inhibition of p53 activity is advantageous to hematopoietic cells upon or after irradiation. An advantage conferred to early progenitors but not mature myeloid cells post-irradiation may account for the delayed rise in the percentages of myeloid cells expressing DDp53 starting at Week 2. As for the MAC1^+^ and B220^+^ lineages, irradiation caused increases in the percentages of DDp53 cells in the CD4^+^ and CD8^+^ T cell lineages (Figure 1C).

**Figure 2:**
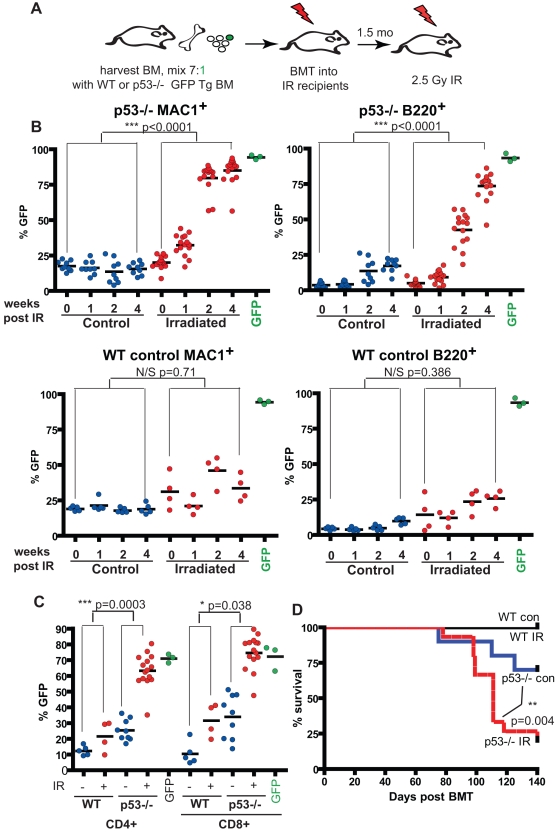
Irradiation selects for *p53*−/− hematopoietic progenitors. Freshly harvested WT BM was mixed with GFP Tg BM (WT control) or GFP Tg *p53*−/− BM at 7∶1 proportions and then transplanted into recipients that had been conditioned with 5 Gy irradiation (schemata in A). Each recipient received a total 8×10^6^ BM cells. Six weeks post-transplantation, five WT control recipients and 14 *p53*−/− recipients were irradiated with 2.5 Gy, while five recipients from the WT control and nine recipients from the *p53*−/− groups were left untreated (control). (B) At 1, 2, and 4 wk post-irradiation peripheral blood was analyzed for the expression of GFP in MAC^+^ myeloid cells and B220^+^ B-cells. (C) At 7 wk post-irradiation, GFP expression in peripheral blood CD4^+^ and CD8^+^ T cells was analyzed. For B and C, the “GFP” control reflects analyses of GFP expression in the indicated lineages for peripheral blood from recipients transplanted with 100% GFP Tg BM. For B and C, *p* values are for *t* tests comparing means of base-line to 4 wk post-irradiation differences in GFP percentages between irradiated and control groups. (D) The same mice were followed for the development of hematopoietic malignancies. Mice were sacrificed when moribund at the indicated times post-BM transplantation (BMT). All sacrificed mice exhibited clear signs of GFP^+^ *p53*−/− thymomas or leukemias. Most mice exhibited greatly enlarged thymi almost entirely composed of CD4^+^CD8^+^ or CD4^+^ GFP^+^ blasted cells, together with infiltration of the spleen. The other sacrificed moribund mice exhibited a leukemic phenotype, with CD4^+^CD8^+^ or CD4^+^ GFP^+^ blast cell infiltration of the spleen and BM but without clear thymic enlargement. Kaplan-Meier curves for lymphoma-free survival are plotted. The *p* value indicates the result of log-rank test analysis.

To confirm these findings using a different model of p53 disruption, we created BM chimeric mice containing cells with null genetic disruption of both *p53* alleles. For these experiments, the null *p53* allele [21] was bred into a transgenic (Tg) line that expresses GFP in all tissues from the Ubiquitin-C promoter [29]. We generated mosaic mice by transplantation of lethally irradiated recipients with WT BM mixed 7∶1 with either *p53*+/+ or *p53*−/− GFP Tg BM. After hematopoiesis was allowed to recover for 6 wk, the mice were sublethally irradiated (2.5 Gy) and competitive hematopoiesis was observed by monitoring peripheral blood over the next 4 wk (Figure 2A). Similar to our results with DDp53 transduced cells, irradiation led to a dramatic selection for *p53*−/− cells in the MAC1^+^ and B220^+^ lineages, resulting in a virtual selective sweep of the p53 mutation within the hematopoietic systems of most recipient animals (Figure 2B). No increase in the percentage of p53 null cells, beyond those expected based on initial ratios transplanted, was evident within unirradiated hematopoiesis.

Irradiation of recipient mice also led to dramatic increases in the percentages of *p53*−/− cells in the CD4^+^ and CD8^+^ T cell lineage (Figure 2C). But in contrast to retrovirally delivered DDp53, we also observed modest selection for *p53*−/− cells in non-irradiated CD4^+^ and CD8^+^ cells, which could either reflect the pre-existence of partially transformed *p53*−/− cells in the T-cell lineage (consistent with eventual T lymphoma development) or skewed selection for *p53*−/− T progenitors in the abnormal thymic environment of irradiated recipient mice.

Irradiated recipients of 7∶1 WT:*p53*−/− BM developed thymomas and T cell leukemias with high penetrance, while development of malignancies in the unirradiated group was significantly reduced and delayed (Figure 2D). All malignancies that developed with or without irradiation expressed GFP and thus were derived from the GFP Tg *p53*−/− donor BM (Figure S2). As expected, recipients of WT BM did not develop thymomas, with or without irradiation, as a single dose of radiation is not sufficient to induce lymphomas within this time frame.

### P53 Disruption Protects Hematopoietic Progenitors from Irradiation-Induced Ablation

**Figure 3:**
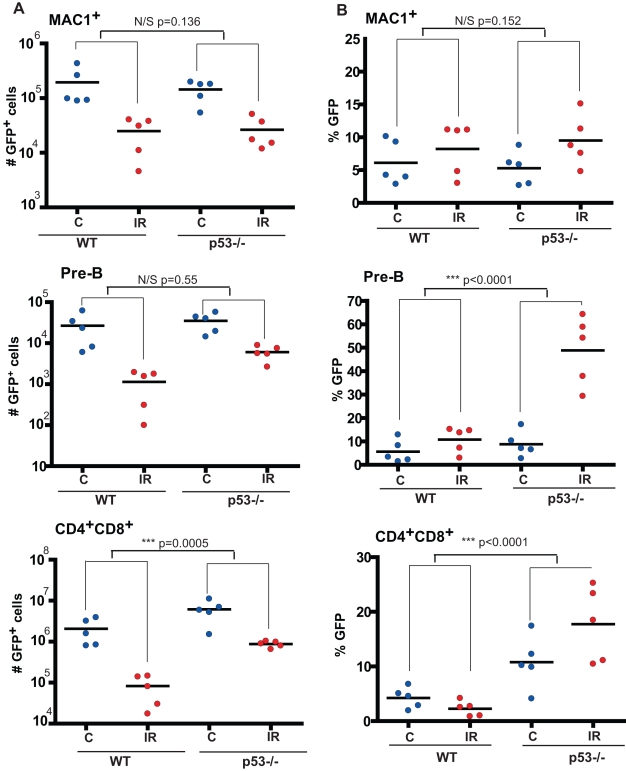
Loss of p53 protects from acute irradiation-induced ablation. BM chimeric mice containing ∼5% GFP Tg BM (WT) or ∼5% GFP Tg *p53*−/− BM were generated using the same experimental procedures described in Figure 2, except that a 19∶1 ratio of WT BM to GFP Tg BM (WT or *p53*−/−) was used. Each recipient received a total 5×10^6^ BM cells. At 48 h post-2.5 Gy sublethal irradiation (IR), the mice were euthanized, and GFP expression was analyzed in the indicated cell populations in the BM and thymi. “C” control mice were not irradiated. (A) The numbers of GFP^+^ Mac1^+^ myeloid cells and GFP^+^ B220^+^CD93^+^CD43^neg^Mac1^neg^ pre-B cells in the BM, and GFP^+^ CD4^+^CD8^+^ DP cells in the thymus, were determined by multiplying the percentage of these subsets among nucleated cells in the BM or thymus times the total number of nucleated cells determined for one tibia or the thymus of each mouse. (B) Percentages of GFP^+^ cells within the indicated lineages are graphed. For (A) and (B), *p* values for *t* tests comparing differences between irradiation-induced changes in GFP percentages or cell numbers between WT and *p53*−*/*− groups are indicated.

To assess whether p53 disruption provides an immediate survival advantage following irradiation, we analyzed hematopoietic tissues at 48 h post-irradiation, focusing on hematopoietic progenitor populations, whose relative survival should determine effects on long-term selection. We used the same experimental design described in Figure 2A, except that a 19∶1 mixture of WT:*p53*−/− GFP BM was transplanted. At 48 h, cells that were killed by direct irradiation-induced damage should have been cleared, while the extent of new proliferation should still be minimal. Irradiation of these chimeric mice at 2.5 Gy had a substantial negative effect on the hematopoietic system, leading to approximately 4-fold reductions in BM cellularity and 2-fold reductions in spleen weight (Figure S3). Yet the impact of irradiation was not equivalent for different hematopoietic lineages. B lineage cells were more radiosensitive than myeloid lineage cells: while BM myeloid cells were reduced in number by about 8-fold, the total BM B220^+^ population was reduced about 30-fold, and BM pro-B and pre-B progenitor populations were reduced 40–60-fold (Figures 3A, S4, and S5A).

Accordingly, irradiation caused substantial increases in the percentages of *p53*−/− cells in the pre-B and pro-B populations (Figures 3B and S5A), as well as in total B220^+^ population (Figure S4). Similar results were obtained at 48 h post-irradiation for recipients of BM where p53 disruption was mediated by expression of DDp53 (unpublished data). Importantly, irradiation reduced the numbers of not only *WT* but also *p53*−/− pre-B and pro-B cells (Figures 3A and S5A). However, the reduction of *p53*−/− B progenitor numbers was clearly less extensive. Therefore, p53 disruption provides partial radioprotection. Examples of flow cytometric profiles for detection of GFP expression in myeloid, pre-B, and pro-B cell populations are shown in Figure S6.

CD4^+^CD8^+^ (double-positive; DP) T cell progenitors in the thymus are known to be very sensitive to irradiation-induced apoptosis [12],[13]. Indeed, at 48 h post-irradiation we observed dramatic ablation of the DP population, while single-positive cells remained relatively unaffected (Figure S7). Similarly to B cell progenitors, disruption of p53 provided a clear radioprotective effect in thymic T-cell progenitors, as irradiation led to increased percentages of *p53*−/− cells in the DP population (Figure 3B). Again, this radioprotection was not absolute: while the numbers of WT GFP^+^ DP cells dropped about 28-fold following irradiation, the numbers of *p53*−/− GFP^+^ DP cells dropped about 7-fold (Figure 3A, right plot). As also observed in experiments shown in Figure 2, for the T cell lineage, p53 loss may confer some advantage even without irradiation, as *p53*−/− percentages increased in DP cells in non-irradiated controls relative to pre-irradiation, and thus the irradiation induced increase in the percentage of GFP^+^ *p53*−*/*− DP cells is less evident than in other lineages (Figure 3B, right plot).

We next examined the effect of irradiation on hematopoietic stem cells (HSC) by examining the HSC-enriched CD150^+^ Lin^neg^/CD48^neg^ BM compartment [30]. In contrast to the lymphoid progenitor pools, CD150^+^ Lin^neg^/CD48^neg^ cell numbers were not affected by irradiation, and we did not observe changes in the percentages of *p53*−/− cells (Figures S5B and S6E). Therefore, disruption of p53 does not appear to provide an immediate survival advantage in HSC pools.

### P53 Disruption Preserves Clonogenic Capacity for Irradiated Hematopoietic Progenitor and Stem Cells

**Figure 4:**
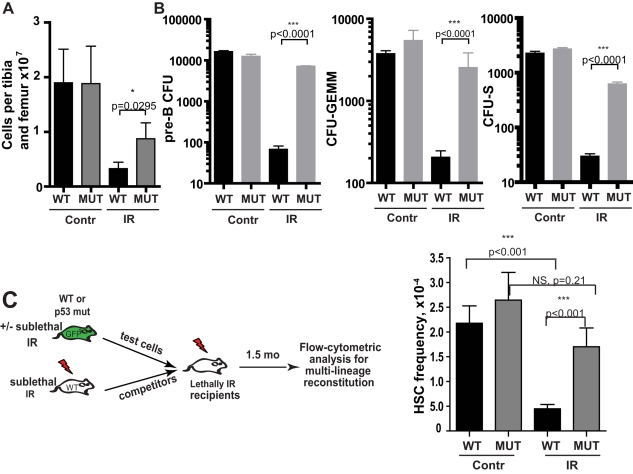
p53 loss protects progenitors from irradiation-induced loss of clonogenic potential. BM was harvested from non-irradiated or irradiated (48 h previously at 2.5 Gy) WT or p53^−/−^ mice and counted in triplicate (A; *n*>5/group). (B) CFU-B, CFU-GEMM, and CFU-S assays were performed as described in Materials and Methods. For CFU-B and CFU-GEMM, colonies were counted 7 or 12 d later, respectively, and the numbers of CFU per mouse (both femurs and tibiae) were calculated. Data reflect experiments performed in triplicate. CFU-S (per both femurs and tibiae of donor mice) were enumerated after 14 d in spleens of recipient mice. Data are combined from two experiments with at least four recipients per group. (C) BM was harvested from GFP-Tg mice that had either been irradiated 48 h before with 2.5 Gy or left untreated (the “test” cells). Different numbers of viable GFP^+^ test cells (0.25×10^4^ to 1×10^5^) were mixed with 10^6^ viable competitor Balb/c (GFP^neg^) BM cells isolated from donors that were irradiated 8 wk prior with 5 Gy. The mixes were injected into lethally irradiated Balb/c recipients. At 3–4 mo post-transplant, peripheral blood was stained with PE-Cy7-anti-Mac1 plus PE-anti-B220 antibodies, and the percentages of GFP^+^ cells in the MAC1^+^ and B220^+^ lineages were determined. The numbers of functional HSC were determined based on the ability of different doses of BM to contribute to hematopoiesis in both the myeloid (Mac1^+^) and B-cell (B220^+^) lineages (with ≥1.0% contribution required to be scored as positive). The numbers of functional HSC were determined with the L-Calc™ software from Stem Cell Technologies and are graphed as HSC frequencies per mouse (both femurs plus both tibiae). Four experiments are combined. *p* values indicate the results of two-tailed ratio of proportions test.

Protection from immediate irradiation-induced ablation does not necessarily correlate with maintenance of long-term proliferative capacity [19]. Therefore, we assessed the impact of p53 disruption on maintenance of clonogenic capacity by progenitor and stem cells. To this end, *p53+/+* or −*/*− mice were irradiated (2.5 Gy) and BM was harvested 48 h later. Note that X-irradiation resulted in a ∼5-fold reduction in BM cellularity by 48 h for WT mice but only ∼2-fold reduction for *p53*−*/*− mice (Figure 4A). BM cells were isolated from *p53+/+* or −*/*− mice 48 h post-irradiation, and either plated in methylcellulose cultures for determination of colony forming units in vitro (CFU-GEMM for granulocytic/erythroid/megakaryocyte/macrophage progenitors or CFU-B for B-lymphoid progenitors) or transplanted into lethally irradiated mice for determination of CFU in spleens (CFU-S, derived from early multipotent progenitors) [31]. Consistent with the analyses above, irradiation resulted in dramatic reductions in CFU-GEMM, CFU-B, and CFU-S numbers (from 20× to 100×; Figure 4B), and p53 disruption provided substantial protection from irradiation-induced elimination of these progenitors (numbers were reduced only 2–3-fold).

To determine the p53-dependent impact of irradiation on numbers of functional HSC, we performed limiting dilution assays. Varying numbers of “test” cells (control or 48 h post-irradiation) were transplanted into lethally irradiated recipient mice, together with a fixed number of competitors to ensure radioprotection. Since irradiation can dramatically reduce the competitive ability of HSC [32], we used competitor BM harvested from previously irradiated donors, in order to ensure that contributions of irradiated “test” HSC are not masked by non-irradiated competitors. In contrast to the lack of irradiation-induced ablation of phenotypically defined HSC (as detected by flow cytometry; Figures S5B and S6E), we observed dramatic reductions in the frequencies of functional WT HSC but not *p53*−*/*− HSC, following irradiation (Figure 4C). Considering the p53-dependence of the effects of irradiation on BM cellularity (Figure 4A), the loss of p53 confers substantial protection from irradiation-induced reductions of functional HSC numbers per mouse.

In summary, the selective advantage for p53 disruption is evident within 48 h in both long- and short-term progenitor populations, supporting a direct role for p53 in radiation-induced cell death. Moreover, beyond preventing the immediate death of stem and progenitor cells following irradiation, the loss of p53 provides an additional selective advantage through protecting clonogenic capacity.

### P53 Disruption Partially Protects Hematopoietic Progenitors from Irradiation-Induced Persistent Reductions of Functional Capacity

Experiments described above demonstrate that loss of p53 protects cells from the acute effects of irradiation by preserving cell survival as measured by phenotypic and functional assays. However, the impact of irradiation is not limited to acute damage. We and others have demonstrated that hematopoietic progenitors suffer from impaired functional capacity long after the acute effects of irradiation have been reversed; i.e., irradiation-induced loss of functional capacity appears to be permanent [32],[33]. We therefore asked whether p53 disruption protects hematopoietic progenitors from this long-term reduction of functional capacity. To this end, we irradiated WT and *p53* null mice, allowed them to recover for 6 wk, and used BM harvested from these mice to set up competitive transplantation experiments with non-irradiated GFP^+^ BM cells at 19∶1 ratios (*p53*−/−:GFP or WT:GFP). By 6 wk post-irradiation, BM cellularity and the numbers of early progenitors are restored [32], and thus these assays measure the impact of p53 disruption on stable reductions of fitness per progenitor caused by irradiation, as opposed to the immediate physical or functional elimination of hematopoietic progenitors.

**Figure 5:**
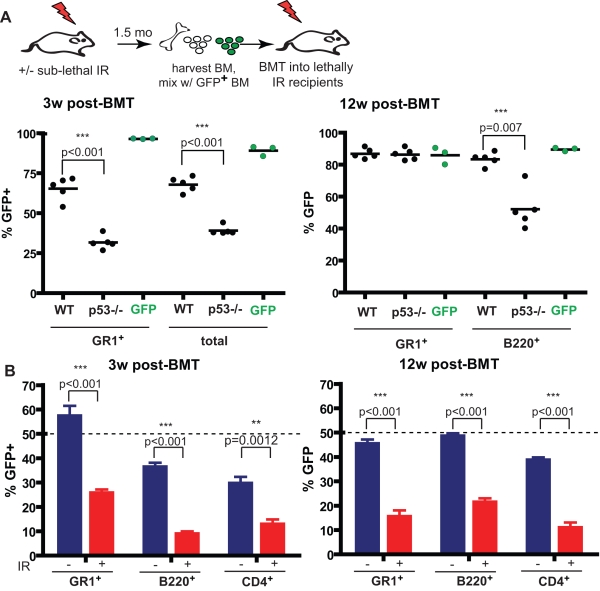
p53 mutation present at the time of X-irradiation provides a long-term fitness advantage during competitive reconstitution. (A) 1.5 mo WT and *p53*−/− mice were irradiated with 5 Gy. Six wk post-irradiation, BM was harvested, mixed with non-irradiated GFP^+^ BM at 1∶19 GFP^+^/WT or GFP^+^/*p53*−/− ratios, and transplanted into lethally irradiated WT recipients. Each recipient received a total of 1×10^7^ BM cells. The animals were bled at 3 and 12 wk post-transplantation and percentages of GFP^+^ cells were determined in either the myeloid lineage (“GR1^+^”) or in total nucleated blood cells (“total”). Note that the *y*-axis for this figure represents contributions from non-irradiated GFP-Tg BM. The “GFP” data points represent analyses of peripheral blood from control recipients of 100% GFP Tg BM (some GFP^neg^ cells are detected even in these recipients, which could reflect residual host cells). (B) Six-week-old GFP^+^ WT and GFP^neg^ *p53*−/− mice were irradiated with 5 Gy or left non-irradiated. 1.5 month post-irradiation, BM was harvested and viable cells were mixed in 50∶50 proportions and transplanted into lethally irradiated recipients to yield two experimental groups (*n* = 5 each): unirradiated GFP^+^ WT plus unirradiated GFP^neg^ *p53*−/− (blue bars) and irradiated GFP^+^ WT plus irradiated GFP^neg^ *p53*−/− (red bars). Peripheral blood was analyzed for GFP expression in the indicated lineages at 3 and 12 wk post-transplantation. The dashed line indicates the expected 50% contribution from GFP Tg hematopoiesis if the fitness of the competing *p53*−/− hematopoiesis were identical. Results are shown for mixes where WT cells are GFP Tg, but similar results were obtained for reciprocal mixes whereby *p53*−/− cells were GFP Tg (unpublished data).

At 3 wk post-transplantation, the percentages of non-irradiated GFP^+^ cells had increased well beyond the initial 5% in the transplanted mixture for both the *p53*−/−:GFP or WT:GFP groups (Figure 5A), reflecting impaired hematopoietic fitness in previously irradiated BM. Still, irradiated *p53*−/− cells fared substantially better than WT cells against unirradiated GFP^+^ competitors as assessed both in the myeloid lineage (GR1^+^) and in total peripheral blood cells (Figure 5A; ∼70% of hematopoiesis was still *p53*−/−). At 12 wk post-irradiation, however, non-irradiated competitors completely took over the myeloid lineage, irrespective of the *p53* status of the irradiated donor BM, as the percent GFP^+^ within the myeloid lineage was indistinguishable from recipients reconstituted with GFP^+^ BM only (“GFP” groups). Still, *p53*−*/*− cells maintained a substantial presence within the B220 lineage, while WT GFP-negative competitors could not be detected.

Thus, loss of p53 failed to completely prevent irradiation-induced loss of fitness of stem/progenitor cells as compared to the fitness of non-irradiated WT cells. However, more relevant to irradiation-induced selection is the selective advantage of mutant cells relative to similarly irradiated WT cells. Therefore, we directly compared the fitness of previously irradiated *p53*−*/*− and WT cells, using an experimental design similar to that used in Figure 5A, but this time using 1∶1 ratios. As controls, we used 1∶1 mixtures of BM isolated from non-irradiated *p53*−*/*− and WT donors. As shown in Figure 5B, previously irradiated *p53*−*/*− BM was clearly more competitive when measured against irradiated WT BM, both in myeloid and lymphoid lineages (Figure 5B, red bars). In contrast, the selective advantage for p53 disruption is much less obvious in recipients of non-irradiated BM mixtures (Figure 5B, blue bars). These results indicate that while loss of p53 is unable to completely protect cells from irradiation-induced loss of fitness, p53 deficiency is still capable of endowing a clear competitive advantage relative to irradiated WT cells. We therefore conclude that in addition to protection from irradiation-induced ablation, protection from persistent loss of fitness contributes to selection for p53-deficientclones by irradiation.

### Disruption of p53 in BM Cells after Recovery from Irradiation Does Not Confer a Durable Selective Advantage

**Figure 6:**
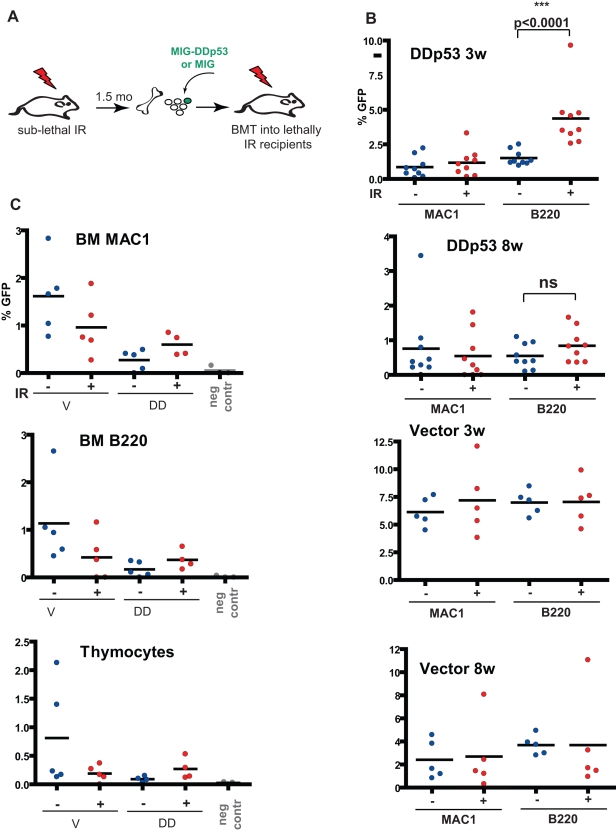
p53 disruption after recovery from the acute effects of irradiation does not provide a selective advantage. (A) BM was isolated from non-irradiated control mice or from mice irradiated 1.5 mo prior with 2.5 Gy and then transduced with MiG-DDp53 (DD) or MiG vector (V). Initial infection efficiency was 2.4% for the V transduced and 1.6% for the DD transduced cells. Transduced cells were injected into lethally irradiated recipient mice, with each recipient receiving 5×10^6^ donor BM cells. (B) Peripheral blood was analyzed for GFP expression in MAC1^+^ and B220^+^ lineages at 3 and 8 wk post-transplantation. Differences for DDp53 Mac1^+^ groups and all Vector groups with or without irradiation were not significant. (C) Recipient mice were sacrificed at 21 wk post-BM transplant, and percentages of GFP^+^ cells determined in the BM and thymus. For BM, GFP representation in the B220^+^Mac1^neg^ (BM B220) or Mac1^+^B220^neg^ (BM MAC1) populations is graphed. For thymus, the percentage of total thymocytes expressing GFP is graphed. The “neg contr” data points are from mice that received no transplantation, which serve as negative controls for GFP detection.

Experiments described above demonstrate that when disrupted at the time of irradiation, p53 loss provides a strong and sustained selective advantage in hematopoietic stem/progenitor cells. We asked whether disruption of p53 is still selectively advantageous when introduced *after* the acute effects of irradiation have been resolved. To address this question, we introduced DDp53 or empty vector into BM progenitors harvested from donors that had been irradiated 6 wk prior to the harvest (or control donors) and transplanted the transduced BM into lethally irradiated recipients (Figure 6A). While transduction efficiency was similar to experiments described in Figure 1, disruption of p53 failed to provide cells with a long-term selective advantage. We consistently observed statistically significant overrepresentation of DDp53 expressing cells in the B-cell lineage at 3 wk post-transplantation (Figure 6B); however, this advantage was no longer apparent at 8 wk post-transplantation. Thus, the observed transient advantage for p53 disruption in the B-cell lineage may reflect an advantage in short-term progenitors that is only evident during the reconstitution phase post-irradiation. Importantly, even at 21 wk post-transplantation, we did not detect increased expansion of DDp53 expressing cells in either non-irradiated or irradiated hematopoiesis, despite the clear presence of a low percentage of GFP^+^ cells in multiple hematopoietic lineages in most recipients (Figure 6C). The continued presence of GFP^+^ cells in multiple lineages more than 4 mo post-transplantation indicates that retroviral delivery of DDp53 did occur in long-term HSC, but that DDp53 expression was not adaptive (i.e., advantageous) within irradiated HSC or more committed progenitors.

We therefore conclude that p53 loss does not provide a selective advantage after the acute effects of irradiation are resolved, and thus selection for p53 mutant clones requires that p53 function is defective at the time of irradiation.

### Competition from Non-Irradiated Cells Blocks the Selective Expansion of Irradiated p53-DeficientClones

The experiments described above argue that the selective advantage of p53 loss at the time of irradiation is attributable to protection of *p53-deficient* progenitors from immediate ablation, loss of clonogenic capacity, and sustained fitness reductions. However, it is possible that selection for p53 deficiency is in part due to additional oncogenic events that are induced by irradiation and whose ability to drive uncontrolled proliferation is permitted by the lack of p53′s critical tumor suppressive function [24]. Should this be the case, then one would expect that once a cell has acquired the ability for uncontrolled proliferation, this clone will expand whether or not competing cells were irradiated.

**Figure 7:**
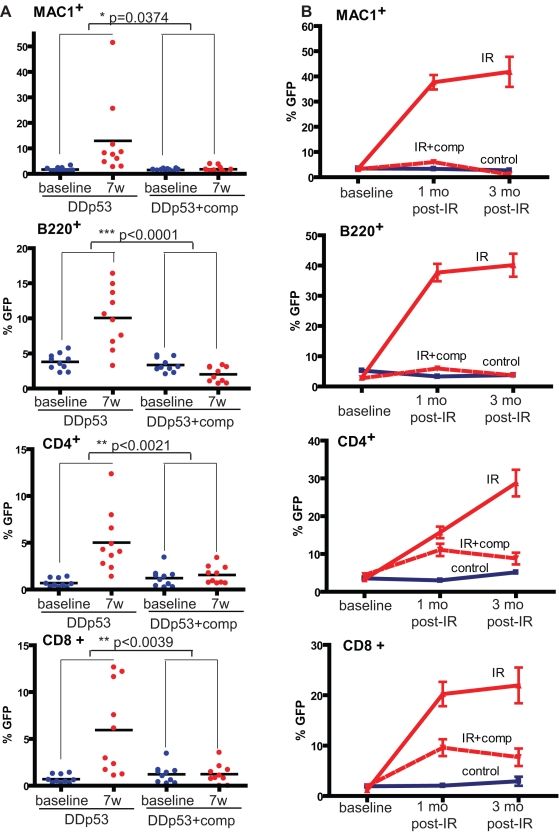
Transplantation of non-irradiated competitors reverses the selective advantage conferred by p53 disruption following irradiation. (A) BM was transduced with MiG-DDp53 and transplanted into 20 lethally irradiated recipients as in Figure 1. Initial infection efficiency was 8.5%. Each of the recipients was transplanted with 1×10^7^ cells. Six weeks post-transplantation, blood was drawn for baseline analysis. Subsequently, the mice were X-irradiated with a single 2.5 Gy dose (IR). Ten of the 20 mice then received transplantation via tail vein injection of 10^7^ whole BM cells (“comp”) from unirradiated mice (within an hour of irradiation). At 7 wk post-sublethal irradiation, peripheral blood was analyzed for the expression of GFP in MAC^+^ myeloid, B220^+^ B lineage, CD4^+^ T lineage, and CD8^+^ T lineage cells. Note that in control experiments, similar transplantation of non-irradiated competitors immediately after 2.5 Gy irradiation resulted in engraftment of transplanted BM, but with maintenance of some host hematopoietis (Figure S8). The indicated *p* values are for *t* tests comparing means of difference in GFP percentages for baseline to 4 wk post-irradiation between irradiated and control groups. (B) BM chimeric mice containing 2.5% GFP Tg *p53*−/− BM were generated as in Figure 3. Each recipient mouse received a total of 4×10^6^ BM cells. Recipients were sublethally irradiated (2.5 Gy; IR) or left unirradiated (no IR) 6 wk after transplantation. One group of 10 irradiated mice (IR+C) then received transplantation via tail vein injection of 10^7^ whole BM cells from unirradiated mice (within an hour post-irradiation). At 1 and 3 mo post-sublethal irradiation, peripheral blood was analyzed for the expression of GFP in the indicated lineages as in (A). For all lineages, GFP percentages either between control and IR groups or between IR and IR+comp groups were statistically significant with *p* values below <0.0001 as determined by two-way ANOVA.

However, experiments presented in Figure 5A argue against this scenario, as unirradiated competitors are capable of effectively outcompeting irradiated *p53* null cells. In these experiments, competition was initiated after recovery from the acute effects of irradiation. To determine the effects of non-irradiated competitors on the acute irradiation-dependent selection for p53 loss, we asked whether non-irradiated competitors, added immediately after irradiation, can counter the selective effect of p53 disruption. To this end, we used an experimental design similar to the one presented in Figure 1. Consistent with results described in Figure 1, DDp53 expression conferred a clear selective advantage in myeloid, T-, and B-cell lineages. In contrast, the addition of non-irradiated competitors completely prevented this selection (Figure 7A).

We performed similar experiments using mice with chimeric *p53*−*/*− hematopoiesis. As expected, irradiation resulted in strong selection for *p53*−/− cells in multiple peripheral blood lineages (Figure 7B). Similar to the results seen with inhibition of p53 by DDp53, non-irradiated competitors potently inhibited this expansion, which is particularly evident in the myeloid and B-cell lineages. As the myeloid lineage is most responsive to changes in HSC pools [34], these data indicate that non-irradiated competitors can reverse selection for p53 disruption within irradiated early progenitor pools. That the effect of competitors on T-lymphoid lineages is more delayed and less dramatic is consistent with the longer half-lives of both mature T cells and their progenitors. We therefore conclude that selection for p53 deficiency by irradiation does not depend on acquisition of additional oncogenic hits in the *p53-deficient* cells.

**Figure 8:**
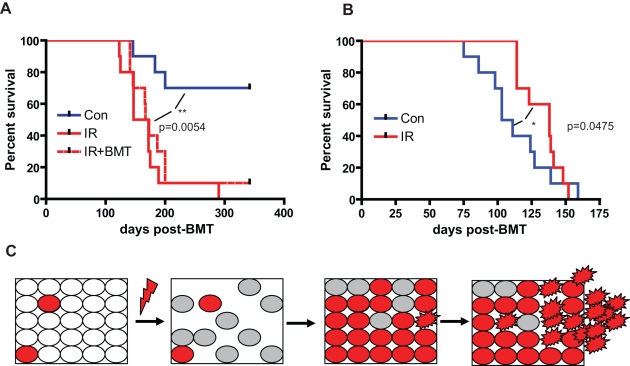
Irradiation promotes *p53*−*/*− lymphomagenesis. (A) BM chimeric mice described in Figure 7B were followed for lymphoma or leukemia development (10 mice/group). Mice were sacrificed when moribund, as in Figure 2D, and leukemia-free survival plotted. Leukemia-free survival differences between irradiated chimeric mice with or without BM transplantation post-irradiation were not significant (*p* = 0.39; logrank test). (B) *p53*−*/*− GFP-Tg BM was transplanted into recipients that had been conditioned with 5 Gy irradiation. Recipients displayed >90% GFP^+^ (*p53*−*/*−) hematopoiesis as assessed 6 wk post-transplant (unpublished data). Six weeks post-transplant, mice were sublethally irradiated (2.5 Gy; IR) or left unirradiated (no IR), and leukemia-free survival is plotted (10 mice/group). Lymphoma/leukemia development was significantly delayed by irradiation (log-rank test). For both (B) and (C), all lymphomas analyzed were CD4^+^/CD8^+^ or CD4^+^ and expressed GFP (indicating their origin from the *p53*−/− GFP Tg BM; Figure S2 and unpublished data). (C) Model: *p53* mutation confers partial resistance to the acute effects of ionizing irradiation and partial protection from the loss of long-term clonogenic potential, leading to selection for hematopoietic cell clones with disrupted p53. Irradiation-induced selection for p53 loss leads to an increased target size for secondary mutations (indicated by altered cell shape), which together with the loss of critical guardian functions of p53 that normally eliminate cells with DNA damage or oncogenic mutations, can drive malignant transformation. The resulting neoplasm may no longer be limited to the confines of normal progenitor cell niches.

We next asked whether inhibiting selection for *p53*−*/*− progenitors by transplantation of non-irradiated competitors translates into reduced incidence of *p53*−*/*− thymomas. The cohorts of recipient mice depicted in Figure 7B were monitored for tumor development. As before (Figure 2D), the irradiation of chimeras containing a minor fraction of *p53*−*/*− hematopoiesis resulted in substantial promotion of *p53*−/− thymoma development (Figures 8A and S2). Surprisingly, transplantation of non-irradiated competitor BM after sublethal irradiation only modestly delayed *p53*−/− thymoma development, and this delay was not statistically significant. Notably, in contrast to the myeloid and B-cell lineages, we observed a substantial delay in the ability of non-irradiated hematopoiesis to displace irradiated *p53*−*/*− T-lineage cells (Figure 7B). Therefore, a large pool of *p53*−*/*− T-progenitors might be maintained for a sufficient period of time to enable the occurrence of transforming secondary oncogenic events, thus underlying the failure of the non-irradiated transplant to effectively prevent thymoma development.

Alternatively, if the causal link between irradiation and tumorigenesis from *p53*−*/*− cells does not involve the selection of radioresistant *p53* null cells, and instead completely relies on the mutagenic action of irradiation, then irradiation should enhance development of T-cell lymphomas in mice with non-chimeric *p53*−*/*− hematopoietic systems. When essentially all hematopoietic cells are *p53*−*/*− mutant, the contribution of selection for p53 null cells toward tumorigenesis should be negated. Thus, we transplanted radio-conditioned recipients with *p53*−*/*− BM and allowed their hematopoietic systems to return to equilibrium (at which point almost all hematopoiesis was donor-derived and thus *p53*−*/*−). We then split the mice into irradiation and control groups and followed the development of lymphomas after irradiation. Contrary to the predicted mutagenic mechanism of irradiation induced tumorigenesis, irradiation not only failed to enhance the development of T-cell malignancies but actually impeded their development, extending the mean survival of the mice (Figure 8B).

In summary, our results demonstrate that irradiation strongly selects for p53-deficientcells in pools of stem and progenitor cells and that this selection does not rely on acquisition of additional oncogenic mutations. These studies instead indicate that altered selection for p53 loss contributes to the causal links between irradiation, p53 disruption, and tumorigenesis.

## Discussion

Dominant paradigms attribute the induction of cancers by DNA-damaging carcinogens (including ionizing radiation) to their mutagenic actions, in that these agents are thought to directly cause activating mutations in proto-oncogenes and loss of function disruption of tumor suppressors [4],[35]. Our data argue that effects of carcinogens on selection of cells with oncogenic lesions, rather than just on their occurrence, need to be considered as well. Specifically, we demonstrate that while p53 loss appears to be selectively neutral within unstressed hematopoiesis, irradiation leads to a potent and sustained selection for cells with disrupted p53 function.

### Multiple Mechanisms Contribute to the Selection of Cells with p53 Disruption

Our results implicate both protection from irradiation-induced ablation and prevention from irradiation-induced loss of clonogenic capacity in the selection for cells that lose p53 function (Figure 8C). The resistance of p53-deficient hematopoietic cells to irradiation-induced apoptosis was shown more than 10 years ago [12]–[16]. However, the relevance of this immediate protection towards long-term selective advantage in competitive contexts has not been directly demonstrated. Indeed, many cell types fail to show p53 dependence for long-term survival upon genotoxic stress despite a clear protection by p53 disruption in short-term assays [19],[36]. Our results demonstrate that in addition to providing a direct survival advantage, loss of p53 also protects hematopoietic progenitors from severe irradiation-induced loss of clonogenic capacity.

This preservation of clonogenic capacity is especially relevant for the HSC compartment. We observe no significant loss of *phenotypic* HSC within 48 h of irradiation, and yet limiting dilution assays indicate that irradiation significantly reduces the numbers of *functional* WT HSC. This reduction is consistent with a recent report that irradiation induces hallmarks of senescence in an HSC-enriched population [33]. In addition to the reduction in numbers of functional stem and progenitor cells, irradiation appears to limit clonal potential per cell. Importantly, the loss of p53 function preserves both the numbers of functional stem/progenitor cells and their fitness (functional capacity as measured in competitive repopulation assays).

### The Role of Selection for Ionizing Irradiation-Induced Tumorigenesis

Homozygous *p53*−*/*− mice are highly prone to spontaneous T-cell lymphomas [20],[21]. While p53 heterozygous mice exhibit much later onset and penetrance of malignancies, they rapidly succumb to lymphomas following irradiation, and these lymphomas invariably lose the WT p53 allele [23]. One possible explanation for the induction of *p53* null lymphomas in *p53* heterozygous mice is that irradiation selects for pre-existing or irradiation induced *p53* loss-of-heterozygosity events, thereby increasing the target size for additional oncogenic mutations that can cooperate with loss of p53 to promote tumor progression. On the other hand, a more prevalent interpretation attributes the carcinogenic effect of irradiation to the induction of new oncogenic mutations [1]. Given the critical importance of p53 in arresting/killing cells with oncogenic mutations [24], many growth-promoting mutations would be expected to synergize with p53 loss in driving abnormal cell expansion and proliferation, in which case the selection for p53 mutation itself might be irrelevant. Indeed, Evan and colleagues demonstrated that conditional activation of p53 during irradiation has no effect on its tumor suppressor function, while activating p53 2 wk after irradiation results in strong suppression of lymphoma development. Since at 2 wk after irradiation the acute damage is resolved, the authors concluded that the essential tumor suppressor function of p53 during irradiation-induced tumorigenesis is to eliminate cells with activated oncogenes, while the p53-dependent elimination of cells with radiation-induced DNA damage is dispensable [37]. Complementary experiments by Donehower and colleagues demonstrated that inactivation of p53 2 wk post-irradiation leads to promotion of lymphomas, virtually indistinguishable from promotion of lymphomas by disruption of p53 prior to irradiation [38], supporting the conclusions reached by Evan and colleagues.

Our studies have reached a different conclusion: p53-dependent elimination of irradiation-damaged cells is actually tumor-promoting. Since irradiation leads to elimination and functional arrest of progenitors with intact p53 function, it selects for p53-deficient clones. Thus, we conclude that the p53-dependent elimination of cells following irradiation *is* important for lymphomagenesis. The critical distinction of our study from those of Evan and Donehower is that we employed models in which *p53* is mutated in only a small fraction (instead of the majority) of cells. This scenario more closely models tumorigenesis induced by irradiation in WT animals. Starting with a small fraction of p53-deficientcells allowed us to observe that while under normal conditions disruption of *p53* is selectively neutral, irradiation endows p53 disrupted cells with a strong selective advantage, driving potent and sustained selection for p53 disrupted cells in stem and progenitor cell pools. The increased fraction of p53-deficientcells should increase the probability that oncogenic events, which are normally cleared through p53-dependent surveillance, would occur in a cell with disrupted p53 function and thus be left unchecked to drive oncogenic transformation. Moreover, the expansion of p53 progenitor clones should further promote lymphoma development by augmenting genetic instability [11],[39]–[41], thus increasing the genetic diversity available to fuel malignant evolution (Figure 8C).

Our results do not support the widely held presumption that direct causation of oncogenic mutations alone is sufficient for the carcinogenic effects of irradiation and instead argue for the importance of selection. First, if direct causation of oncogenic mutations were the only mechanism of irradiation-induced oncogenesis, one would expect that irradiation should boost lymphomagenesis in recipients of 100% *p53*−/− BM, as oncogenic mutations (normally eliminated through p53-dependent surveillance mechanisms) would be able to drive malignant progression. When the majority of hematopoietic cells are *p53*−/−, the contribution of selection for *p53* null cells is negated, while the contributions of induced oncogenic mutations (normally eliminated dependent on p53) should be maximal. Contrary to the predominant role of mutagenesis and consistent with the importance of selection, we observed that irradiation actually delayed lymphoma development in mice with mostly *p53*−/− hematopoiesis (Figure 8B). Second, if the irradiation-induced lymphomas in *p53*−/− chimeras were solely dependent on the induction of oncogenic mutation, cooperating with p53 loss, these mutations would be expected to drive abnormal proliferation regardless of the status of competitor cells. However, this is not the case: addition of non-irradiated competitor BM dramatically inhibits the selective expansion of p53-deficientcells (Figure 7). Still, irradiation does accelerate tumor development in *p53*−/− mice less than 7 d old (but not in adult *p53*−/− mice) [23], as well as in the mouse model used by the Donehower group [38]. Moreover, co-transplantation of unirradiated BM failed to prevent irradiation-promoted lymphomagenesis despite strongly inhibiting selection for p53 mutant cells (Figure 8A), although the inhibition of selection was substantially delayed in the T-cell lineage (Figure 7B). Therefore, in some contexts irradiation can promote p53-deficient tumorigenesis independent of altered selection for p53 loss.

### Additional Evidence for the Importance of Selection in Carcinogenesis

Ultraviolet (UV) light exposure has been shown to increase the numbers and size of *p53* mutant clones in human skin [42] and to induce the expansion of *p53* mutant/Ras activated premalignant cells in organotypic skin cultures [43]. The expansion of *p53* disrupted clones in mouse skin required continued UV-B exposure [44], contrasting with the stable selection for p53 disruption following a single exposure to X-irradiation in our studies. Of interest, conferring increased apoptosis resistance to skin cells actually hampers the expansion of *p53*−*/*− clones and the frequency of UV-induced skin cancers in mice [45]. Of course, selection for p53 loss is not limited to contexts of initiation. Within established tumors, chemotherapy leading to DNA damage and anti-angiogenic therapy leading to hypoxia have each been shown to potently select for *p53* disruption [24],[46],[47].

Direct links between carcinogen exposure and causation of oncogenic mutations have clearly been implicated in some contexts. For example, the skin cancer-associated mutational spectra in *INK4A* and *p53* genes are specific to UV light-induced mutagenesis [48]–[50]. On the other hand, the presence of an initiating mutation is not sufficient for tumorigenesis unless the mutation leads to clonal expansion, as the small target size of an unselected mutant clone should substantially limit the chances for acquisition of additional oncogenic events. Thus, even for UV irradiation, where the evidence for direct causation of oncogenic mutations is the strongest, both the mutagenic and selective functions appear to be implicated in UV-induced carcinogenesis. Notably, genetic alterations unique to ionizing radiation are not evident in cancers associated with radiation exposure [1].

Of note, the loss of p53 is not the only oncogenic event that can be selected by irradiation. While in the context of previous irradiation p53 inhibition failed to provide a stable selective advantage (Figure 6), cells that expressed the Notch1 mutant ICN were strongly selected for within previously irradiated progenitor cell pools [32]. Notably, co-transplantation of non-irradiated hematopoiesis potently inhibits both selection for ICN expressing cells and the resulting leukemogenesis. The ability of healthy WT competitors to limit expansion of oncogenically mutated cells has also been demonstrated in other contexts of genetic or chemical impairment of cell proliferation [51],[52]. Thus, the expansion of an initiated clone requires both conditions of reduced fitness within a progenitor pool and the presence of cells with oncogenic mutations adaptive or resistant to the particular fitness-reducing context.

In summary, while current paradigms primarily focus on how mutations in key genes controlling cell proliferation and survival contribute to the evolution of cancer, the data presented here, together with previous studies, indicate that greater focus on how carcinogenic contexts impact on selection of cells with oncogenic events such as p53 disruption may be critical for understanding, preventing, and perhaps even treating cancers.

## Materials and Methods

### Ethics Statement

The University Colorado Denver School of Medicine Animal Care and Use Committee approved all mouse experiments.

### Retroviral Constructs and Infections

MiG constructs expressing DDp53 (“dimerization domain” of p53) have been previously described [51]. Viral particles were assembled using ψNX-Eco packaging cells as previously described [51]. Freshly isolated BM cells were transduced with retrovirus containing ψNX-Eco supernatants in non-adhesive six-well plates using the spin-fection technique (centrifugation at 910 g for 1.5 h in the presence of 8 μg/ml polybrene). Cells were then washed once with PBS and transplanted into recipient mice.

### BM Transplantations and Irradiation

Mice were starved the night before irradiation to reduce intestinal irradiation damage. BM transplant recipients were lethally irradiated with two 5 Gy doses separated by 3–4 h (10 Gy combined) using an X-ray source (RadSource RS2000 irradiator). A single 2.5 Gy dose was used for sublethal irradiation. Donor BM was transplanted via tail vein injections.

### CFU Assays

BM was harvested from the femurs and tibiae of non-irradiated or irradiated (48 h previously at 2.5 Gy) WT or p53^−/−^ Balb/c donors. Live cells were counted by propidium iodide exclusion using the Cell Lab Quanta SC cytometer (in triplicate). Pre-B CFU (CFU-B) and CFU-GEMM assays were performed with M3630 and M3434 media (Stem Cell Technologies, Vancouver, British Columbia, Canada), respectively, according to the manufacturer's instructions. Colonies were counted 7 d (CFU-B) or 12 d (CFU-GEMM) later, and the numbers of CFU per mouse (both femurs and tibiae) were calculated. For CFU-S, recipient mice were irradiated to sufficiently suppress endogenous CFU-S development (7.5 Gy; unpublished data) and then transplanted with varied numbers (6×10^4^–1.3×10^6^) of live cells. Recipients were euthanized 14 d later, and spleens were harvested and fixed in Bouin's fixative overnight. CFU-S were enumerated the following day and the number of CFU-S per mouse was calculated based on the number of cells that had been harvested from each donor. Several recipients of irradiated WT BM died prior to spleen harvest, presumably due to hematopoietic failure.

### Mice

Balb/c mice were purchased from the National Cancer Institute or generated by in-house breedings. *p53*−/− mice [21] were purchased from Jackson Labs. GFP Tg mice were the generous gift of the Kappler/Marrack lab [29]. GFP Tg and *p53*−/− animals were backcrossed together into the Balb/c background for 10−11 generations.

### Tissue Harvests and Flow Cytometric Analyses

Single-cell suspensions of hemolysed BM, spleen, or peripheral blood were washed in PBS containing 1% BSA (BSA-PBS) and resuspended in BSA-PBS plus 5% supernatant from hybridoma cells producing the 2.4G2 monoclonal antibody against the Fc receptor (to block Fc receptors on hematopoietic cells, which nonspecifically bind antibodies). 10^5^ to 10^6^ cells were stained in 20 µl of antibody solution (1∶200 dilution of each antibody) for 30 min on ice. Cells were washed once with 1 ml of BSA-PBS and resuspended in 400 µl of BSA-PBS for flow cytometric analysis. The following PharMingen (San Diego, California, USA) antibodies against mouse proteins were used: phycoerythrin (PE)-linked anti-B220, PE-anti-Ter119, PE-anti-GR-1, PE-anti-CD3, PE-anti-CD4, and PE-anti-CD8 (together, these PE-linked antibodies constitute the “Lin” stain to gate out lineage committed progenitors and mature cells), and allophycocyanin (APC)-linked anti-B220. PE-Cy7-linked streptavidin, biotin-anti-CD93 (AA4.1), PE-Cy7-anti-CD11b (Mac1), and PE-anti-CD48 were purchased from eBioscience (San Diego, California, USA). Pacific Blue-linked streptavidin was purchased from Invitrogen (Carlsbad, California, USA), and APC-anti-CD150 was from BioLegend (San Diego, California, USA). Fluorescence was detected with CyAn (DAKO, Carpinteria, California, USA) or Cell Quanta SC MPL (Beckman Coulter, Allendale, New Jersey, USA) cytometers. Automated haematocrit analysis of peripheral tail vein blood was performed using a Cell-Dyn 1700 System (Abbott, Abbott Park, Illinois, USA).

### Analyses of Lymphoma and Leukemia Development

Transplanted mice were monitored for disease development, as judged by increasing percentages of GFP^+^ cells (invariably CD4^+^/CD8^+^ or CD4^+^) with blast morphology in peripheral blood of transplanted animals, as well as symptoms, such as reduced mobility, hunching, and labored breathing. Moribund animals were sacrificed and examined for thymoma or leukemia development, as well as the dissemination of the lymphoma to spleen and BM. The expression of lineage markers (CD4, CD8, B220, and Mac1) on GFP^+^ leukemia and lymphoma cells was assessed by antibody staining and flow cytometry.

### Statistical Analysis

Statistical analyses were performed using Prizm 4 software from GraphPad. GFP percentage data were transformed with the arcsine transformation prior to analysis. Unless otherwise specified, *p* values represent the results of unpaired two-tailed *t* tests. In cases when an *F* test indicated different variances between the groups, Welsh's correction was applied. Kaplan-Meier survival curves were used to calculate statistical significance for differences in leukemia development (using the Logrank test). Limiting dilution analysis for the calculation of HSC frequencies and statistical significance was performed using the L-Calc software package from Stem Cells. All presented data are representative of at least two independent experiments. N/S indicates *p* values greater than 0.05, * between 0.05 and 0.01, ** between 0.01 and 0.001, and *** below 0.001.

## Supporting Information

Figure S1 **Examples of flow cytometric gating strategies for determination of GFP expression in specific lineages in peripheral blood.** (A) Non-irradiated DDp53 mosaics. (B) Irradiated DDp53 mosaics, 2 wk post-irradiation.
(0.03 MB PDF)

Figure S2 **Lymphomas and leukemias that develop in p53−/−GFP Tg:WT chimeras are from p53−/−GFP^+^ donor BM.** Mice from the experiments shown in Figures 2 and 8 were followed for the development of hematopoietic malignancies. All sacrificed mice exhibited clear signs of thymomas or leukemias. Mice exhibited greatly enlarged thymi and/or spleens almost entirely composed of GFP^+^ blasted cells (either CD4^+^CD8^+^ or CD4^+^). An example of flow cytometric analysis of cells from the thymus, peripheral blood, and BM of a moribund mouse in the IR group (from the experiment presented in Figure 8A) is shown. The CD4^+^CD8^+^ GFP^+^ lymphoma in this example constitutes the majority of cells in the thymus and peripheral blood but only a small fraction of BM cells (typical of a lymphoma). All other sacrificed moribund mice exhibited a similar development of GFP^+^ lymphomas or leukemias.
(0.35 MB PDF)

Figure S3 **Irradiation reduces spleen and BM cellularity.** Mice transplanted with MiG (Vector) or MiG-DDp53 transduced BM (as in Figure 1) were sublethally irradiated 6 wk after BM transplantation and sacrificed 48 h later. Spleen weights and tibia cellularity were determined.
(0.02 MB PDF)

Figure S4 **X-irradiation reduces the numbers of BM B220^+^ cells, leading to selection of p53−/− cells.** BM chimeric mice from the experiments described in Figure 3, containing about 5% GFP Tg BM (WT) or 5% GFP Tg *p53*−/− BM, were killed 48 h post-irradiation and analyzed. Left: numbers of B220^+^ cells per one tibia; right: percentage of GFP^+^ cells among B220^+^ lineage. Statistical analyses were performed as in Figure 3.
(0.02 MB PDF)

Figure S5 **Irradiation results in selection for p53 mutation in pro-B cell pools, but not phenotypic HSC-enriched pools, within 48 h.** BM chimeric mice were from the experiments described in Figure 3, containing about 5% GFP Tg BM (GFP) or 5% GFP Tg *p53*−/− BM. At 48 h post-2.5 Gy irradiation, the mice were euthanized, and GFP expression in the BM was analyzed in the indicated populations by antibody staining and flow cytometry: (A) pro-B cell pools (B220^+^CD93^+^CD43^+^Mac1^neg^) and (B) HSC pools (Lin^neg^CD48^neg^CD150^+^). Percentages and numbers of GFP^+^ cells within the indicated lineages are graphed. Statistical analyses were performed as in Figure 3.
(0.02 MB PDF)

Figure S6 **Examples of flow cytometric gating strategies for the analyses of pro-B, pre-B, myeloid, and HSC-enriched populations in the BM.** Gating strategies are shown for the quantitation of GFP^+^ cells within the indicated populations used for Figures 3 and S5. (A) WT chimeric BM without irradiation (mock). (B) WT chimeric BM, 48 h post-irradiation. (C) *p53*−/− chimeric BM without irradiation. (D) *p53*−/− chimeric BM, 48 h post-irradiation. For (A–D), the percentages of GFP^+^ and GFP^neg^ gates within the myeloid (Mac1^+^), pre-B (B220^+^CD93^+^CD43^neg^Mac1^neg^), and pro-B (B220^+^CD93^+^CD43^+^Mac1^neg^) cell compartments are indicated. (E) Examples of flow profiles and gating strategies for the Lin^neg^CD48^neg^CD150^+^ HSC-enriched population. The percentages of HSC-enriched cells (elliptical R3 gate, relative to total live cell gate) and of GFP^+^ cells within the HSC-enriched gate are indicated. Arrows indicate the gating strategy.
(0.25 MB PDF)

Figure S7 **Irradiation selectively ablates the CD4^+^CD8^+^ double-positive population in the thymus.** Thymocytes from mice described in Figure 3 were stained with antibodies against CD4 and CD8 and analyzed by flow cytometry. Representative flow profiles are shown, with percentages of cells in sub-populations indicated.
(0.03 MB PDF)

Figure S8 **Transplantation of BM after 2.5 Gy irradiation results in chimeric engraftment.** Balb/c mice (*n* = 5) were irradiated at 2.5 Gy and then transplanted with 10^7^ whole BM cells from a GFP-Tg donor mouse. Peripheral blood was analyzed 5 mo later for GFP^+^ cell contributions to myeloid, B-cell, and T-cell lineages. GFP percentages were less than 1% in all negative controls (untransplanted Balb/c mice), and the percent GFP^+^ within the B220^+^, Mac-1^+^, CD4^+^, and CD8^+^ gates were 86.9%, 98.5%, 90.2%, and 92.8%, respectively, from a GFP Tg mouse (the positive control for GFP detection), indicating that significant GFP^neg^ hematopoiesis was detected in recipient mice.
(0.01 MB PDF)

## Abbreviations

BM: bone marrow
DP: double-positive
HSC: hematopoietic stem cells
MiG: MSCV-ires-GFP retroviruses
Tg: transgenic
UV: ultraviolet
WT: wild-type
