# Supplementary figures and images for: Irradiation Selects for p53-Deficient Hematopoietic Progenitors
Source: PLoS Biol. 2010 Mar 2;8(3):e1000324. doi: 10.1371/journal.pbio.1000324 (PMC2830447; doi:10.1371/journal.pbio.1000324)

Figure S1

A

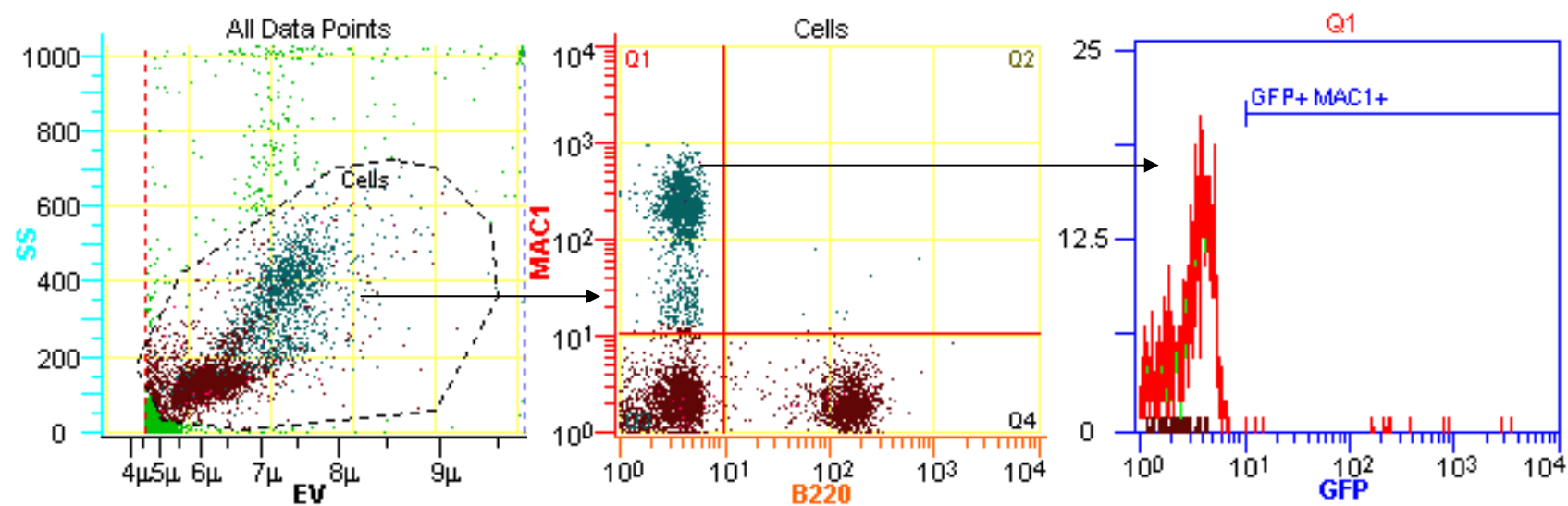

B

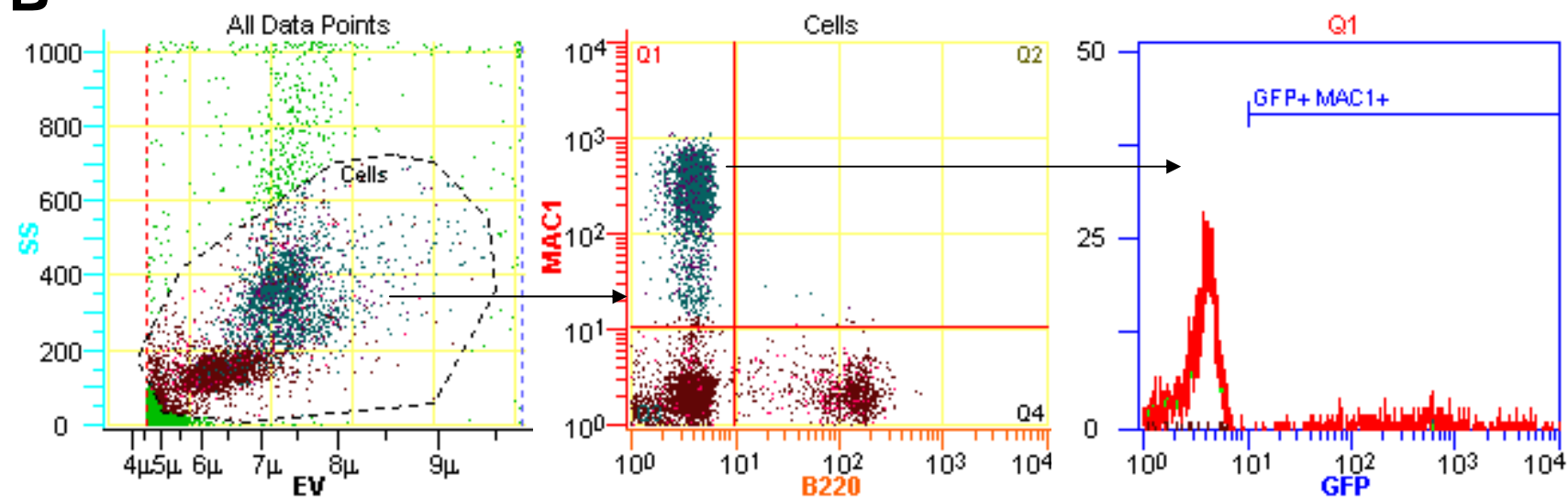

Supplement: Figure S1 — Examples of flow cytometric gating strategies for determination of GFP expression in specific lineages in peripheral blood. (A) Non-irradiated DDp53 mosaics. (B) Irradiated DDp53 mosaics, 2 wk post-irradiation. (0.03 MB PDF) [file pbio.1000324.s001.pdf]

Figure S2

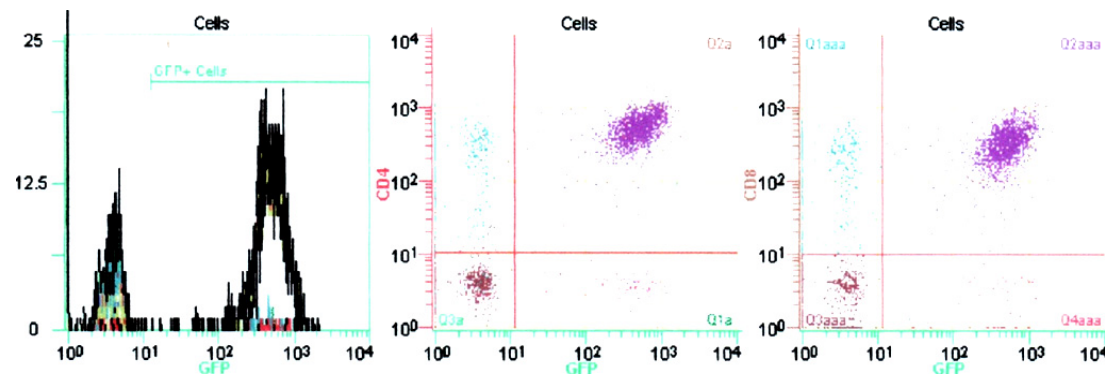

Thymus

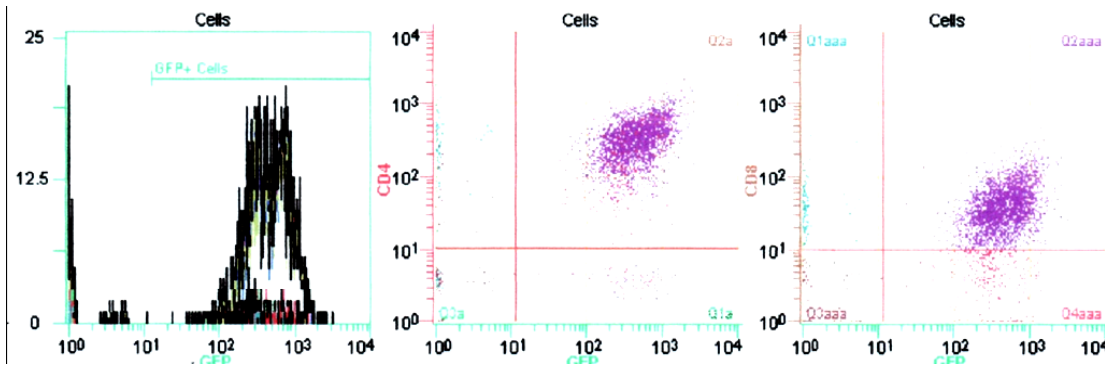

Peripheral Blood

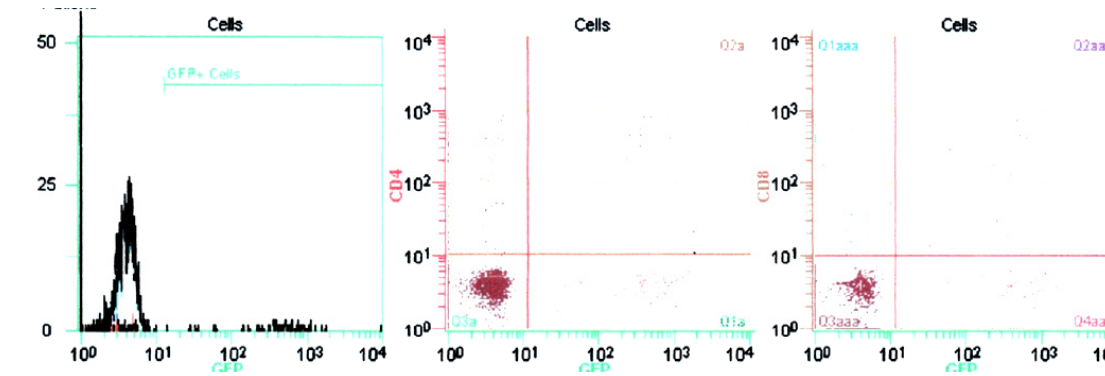

Bone Marrow

GFP →

Supplement: Figure S2 — Lymphomas and leukemias that develop in p53−/−GFP Tg:WT chimeras are from p53−/−GFP+ donor BM. Mice from the experiments shown in Figures 2 and 8 were followed for the development of hematopoietic malignancies. All sacrificed mice exhibited clear signs of thymomas or leukemias. Mice exhibited greatly enlarged thymi and/or spleens almost entirely composed of GFP+ blasted cells (either CD4+CD8+ or CD4+). An example of flow cytometric analysis of cells from the thymus, peripheral blood, and BM of a moribund mouse in the IR group (from the experiment presented in Figure 8A) is shown. The CD4+CD8+ GFP+ lymphoma in this example constitutes the majority of cells in the thymus and peripheral blood but only a small fraction of BM cells (typical of a lymphoma). All other sacrificed moribund mice exhibited a similar development of GFP+ lymphomas or leukemias. (0.35 MB PDF) [file pbio.1000324.s002.pdf]

## Figure S3

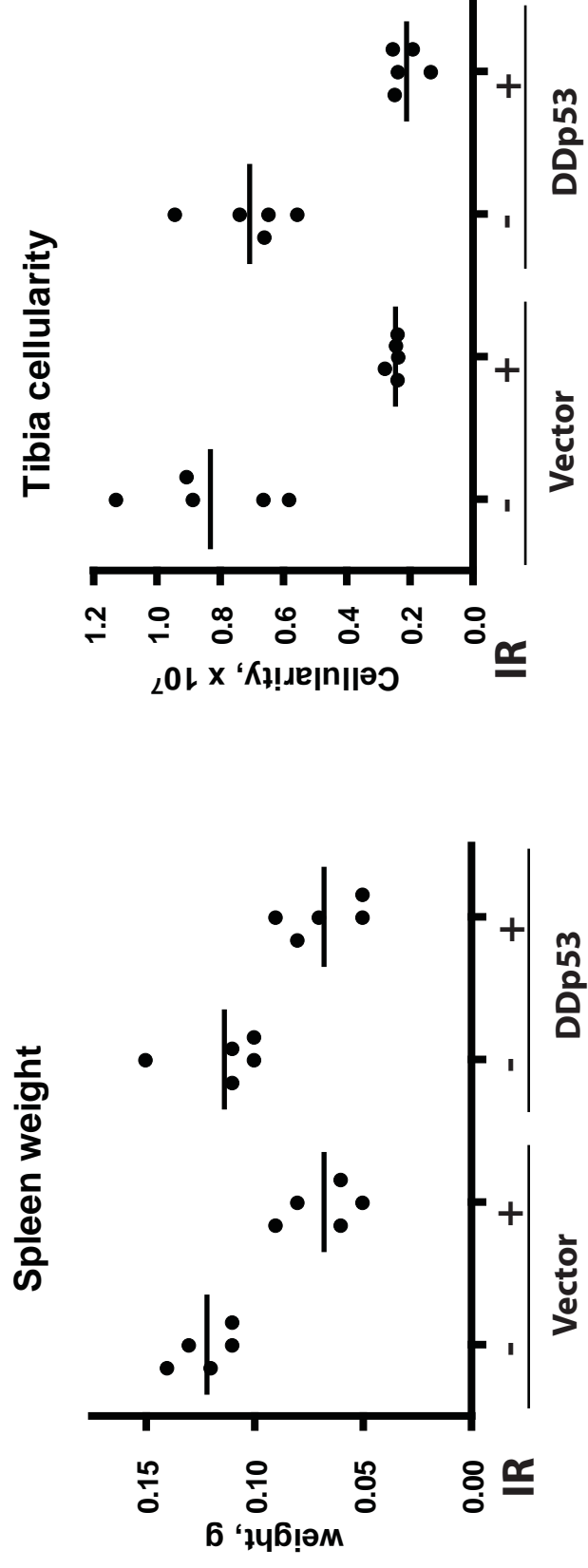

Supplement: Figure S3 — Irradiation reduces spleen and BM cellularity. Mice transplanted with MiG (Vector) or MiG-DDp53 transduced BM (as in Figure 1) were sublethally irradiated 6 wk after BM transplantation and sacrificed 48 h later. Spleen weights and tibia cellularity were determined. (0.02 MB PDF) [file pbio.1000324.s003.pdf]

**Figure S4**

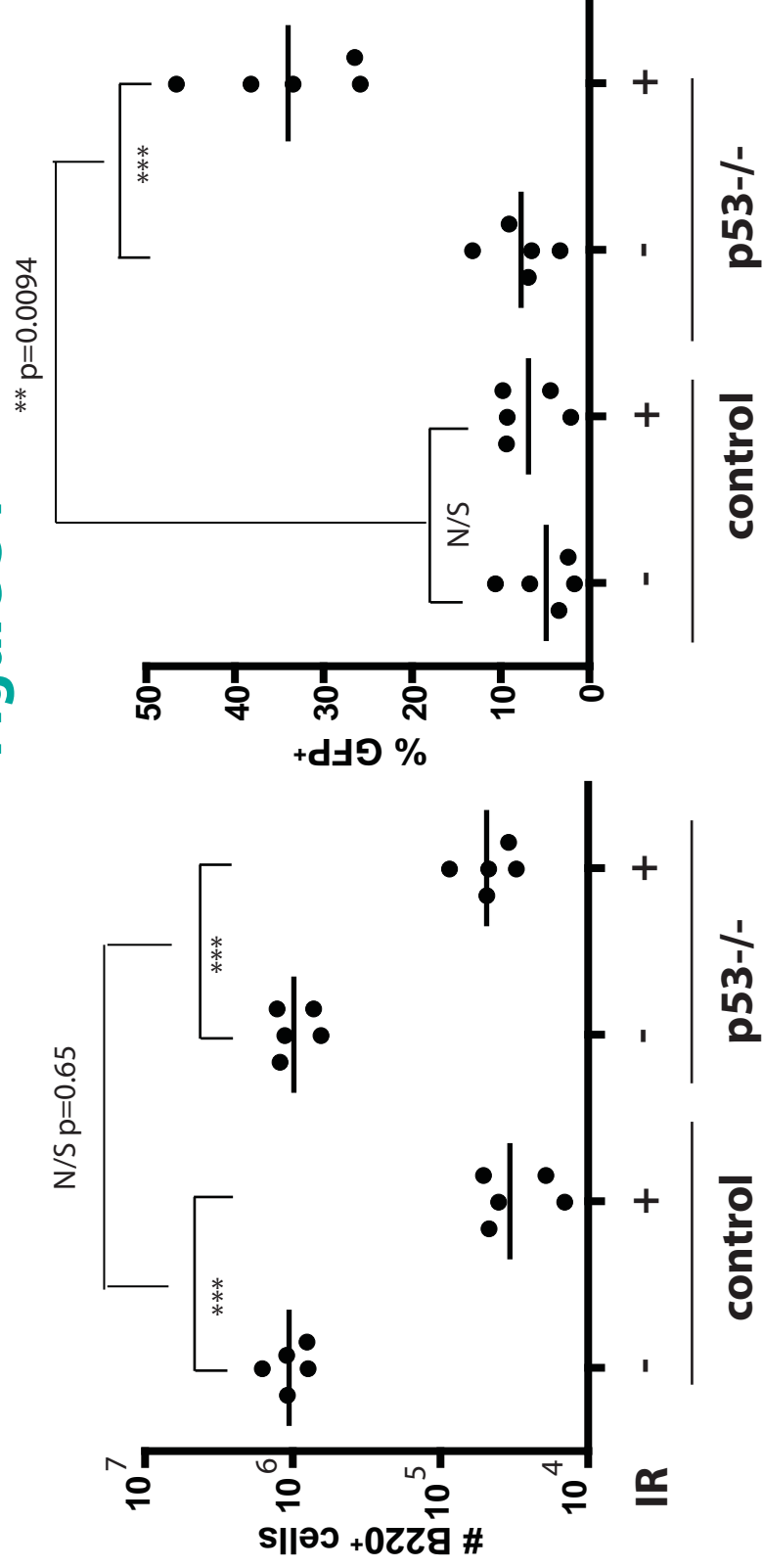

Supplement: Figure S4 — X-irradiation reduces the numbers of BM B220+ cells, leading to selection of p53−/− cells. BM chimeric mice from the experiments described in Figure 3, containing about 5% GFP Tg BM (WT) or 5% GFP Tg p53−/− BM, were killed 48 h post-irradiation and analyzed. Left: numbers of B220+ cells per one tibia; right: percentage of GFP+ cells among B220+ lineage. Statistical analyses were performed as in Figure 3. (0.02 MB PDF) [file pbio.1000324.s004.pdf]

Figure S5

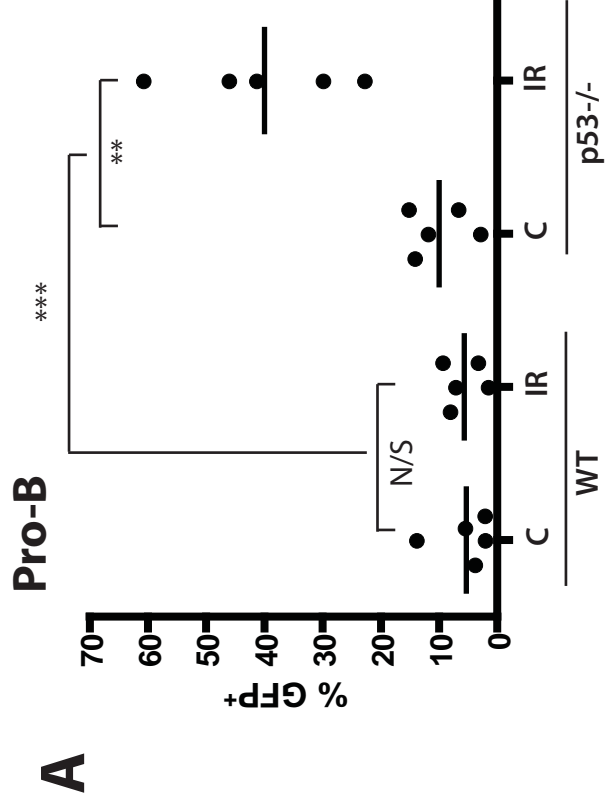

**B**

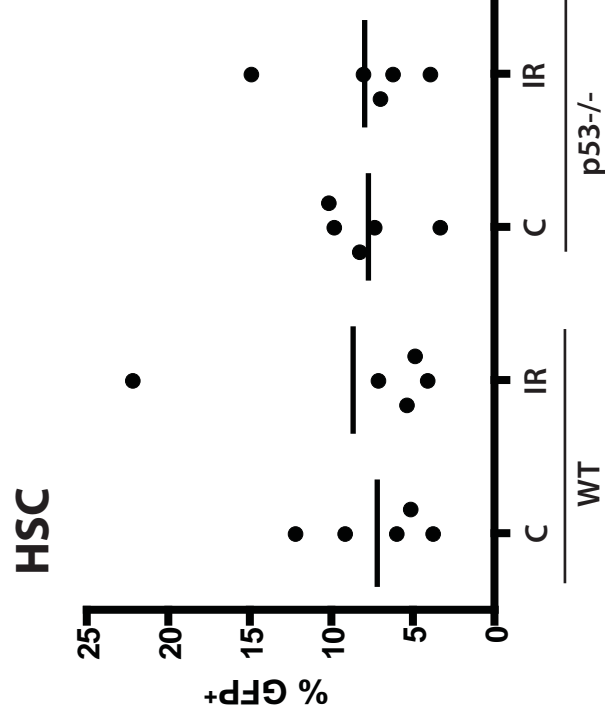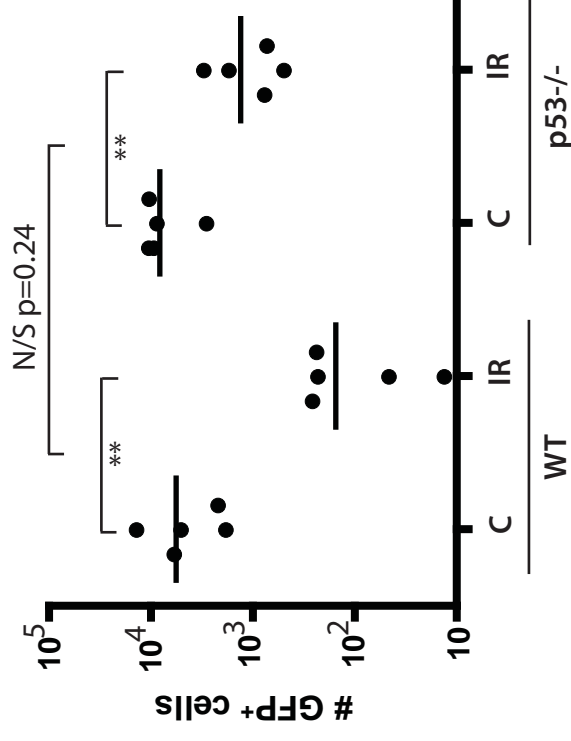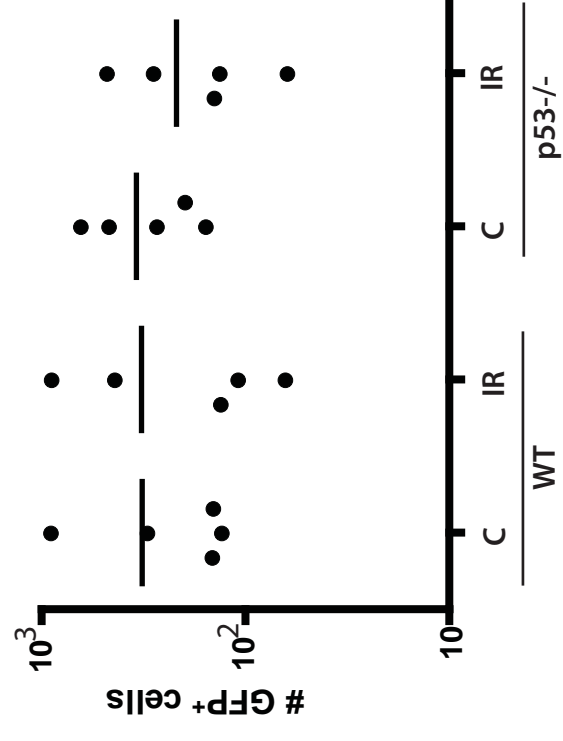

Supplement: Figure S5 — Irradiation results in selection for p53 mutation in pro-B cell pools, but not phenotypic HSC-enriched pools, within 48 h. BM chimeric mice were from the experiments described in Figure 3, containing about 5% GFP Tg BM (GFP) or 5% GFP Tg p53−/− BM. At 48 h post-2.5 Gy irradiation, the mice were euthanized, and GFP expression in the BM was analyzed in the indicated populations by antibody staining and flow cytometry: (A) pro-B cell pools (B220+CD93+CD43+Mac1neg) and (B) HSC pools (LinnegCD48negCD150+). Percentages and numbers of GFP+ cells within the indicated lineages are graphed. Statistical analyses were performed as in Figure 3. (0.02 MB PDF) [file pbio.1000324.s005.pdf]

Figure S7

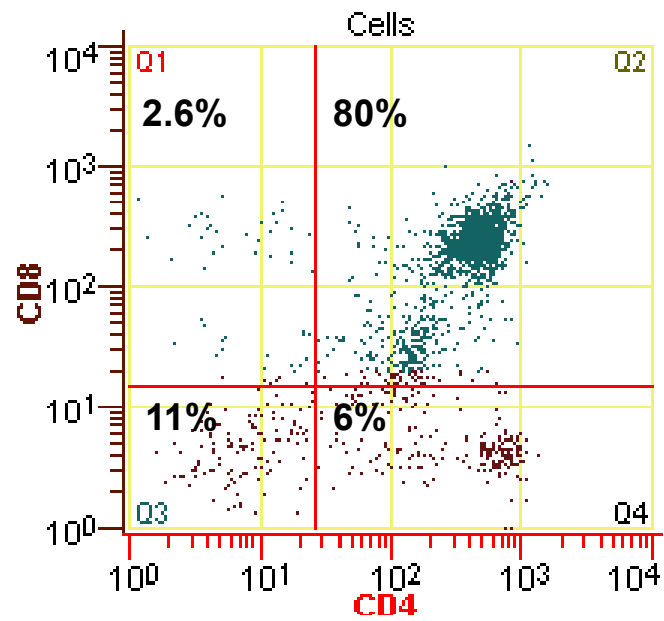

Control

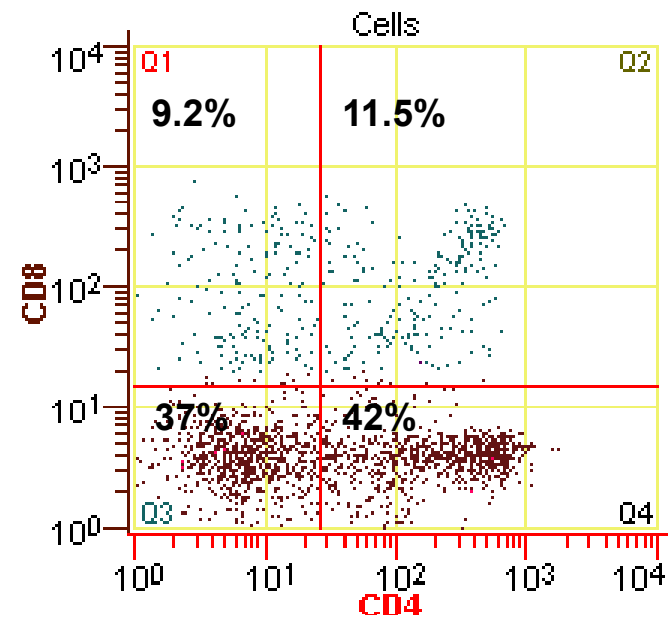

Irradiated

Supplement: Figure S7 — Irradiation selectively ablates the CD4+CD8+ double-positive population in the thymus. Thymocytes from mice described in Figure 3 were stained with antibodies against CD4 and CD8 and analyzed by flow cytometry. Representative flow profiles are shown, with percentages of cells in sub-populations indicated. (0.03 MB PDF) [file pbio.1000324.s007.pdf]

Figure S8

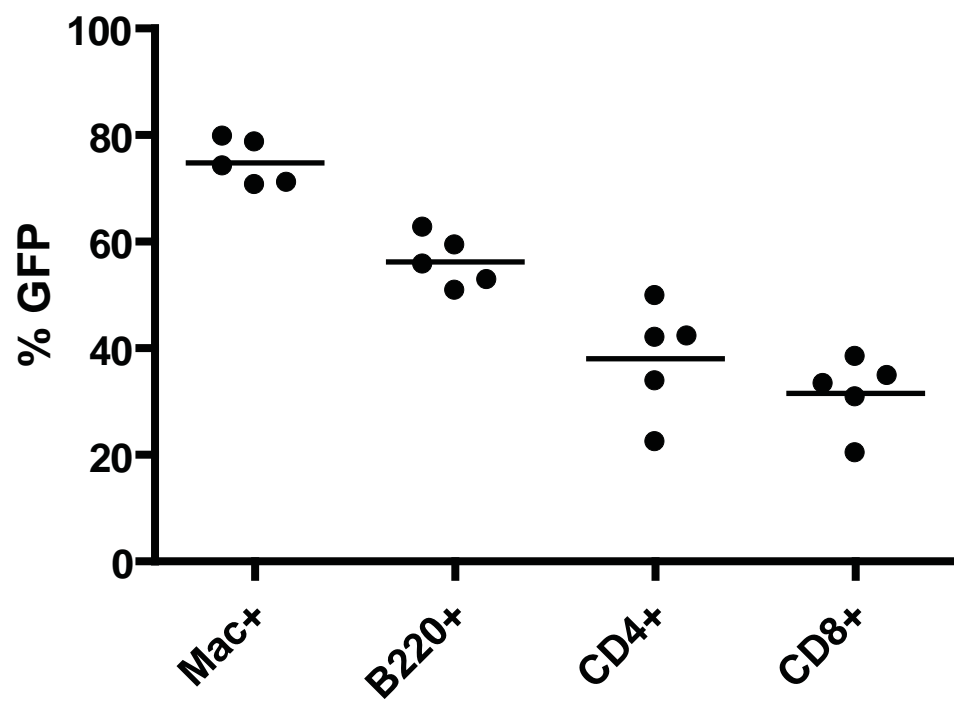

Supplement: Figure S8 — Transplantation of BM after 2.5 Gy irradiation results in chimeric engraftment. Balb/c mice (n = 5) were irradiated at 2.5 Gy and then transplanted with 107 whole BM cells from a GFP-Tg donor mouse. Peripheral blood was analyzed 5 mo later for GFP+ cell contributions to myeloid, B-cell, and T-cell lineages. GFP percentages were less than 1% in all negative controls (untransplanted Balb/c mice), and the percent GFP+ within the B220+, Mac-1+, CD4+, and CD8+ gates were 86.9%, 98.5%, 90.2%, and 92.8%, respectively, from a GFP Tg mouse (the positive control for GFP detection), indicating that significant GFPneg hematopoiesis was detected in recipient mice. (0.01 MB PDF) [file pbio.1000324.s008.pdf]
