# Supplementary material for: Irradiation Selects for p53-Deficient Hematopoietic Progenitors
Source: PLoS Biol. 2010 Mar 2;8(3):e1000324. doi: 10.1371/journal.pbio.1000324 (PMC2830447; doi:10.1371/journal.pbio.1000324)

WT mock

Figure S6A

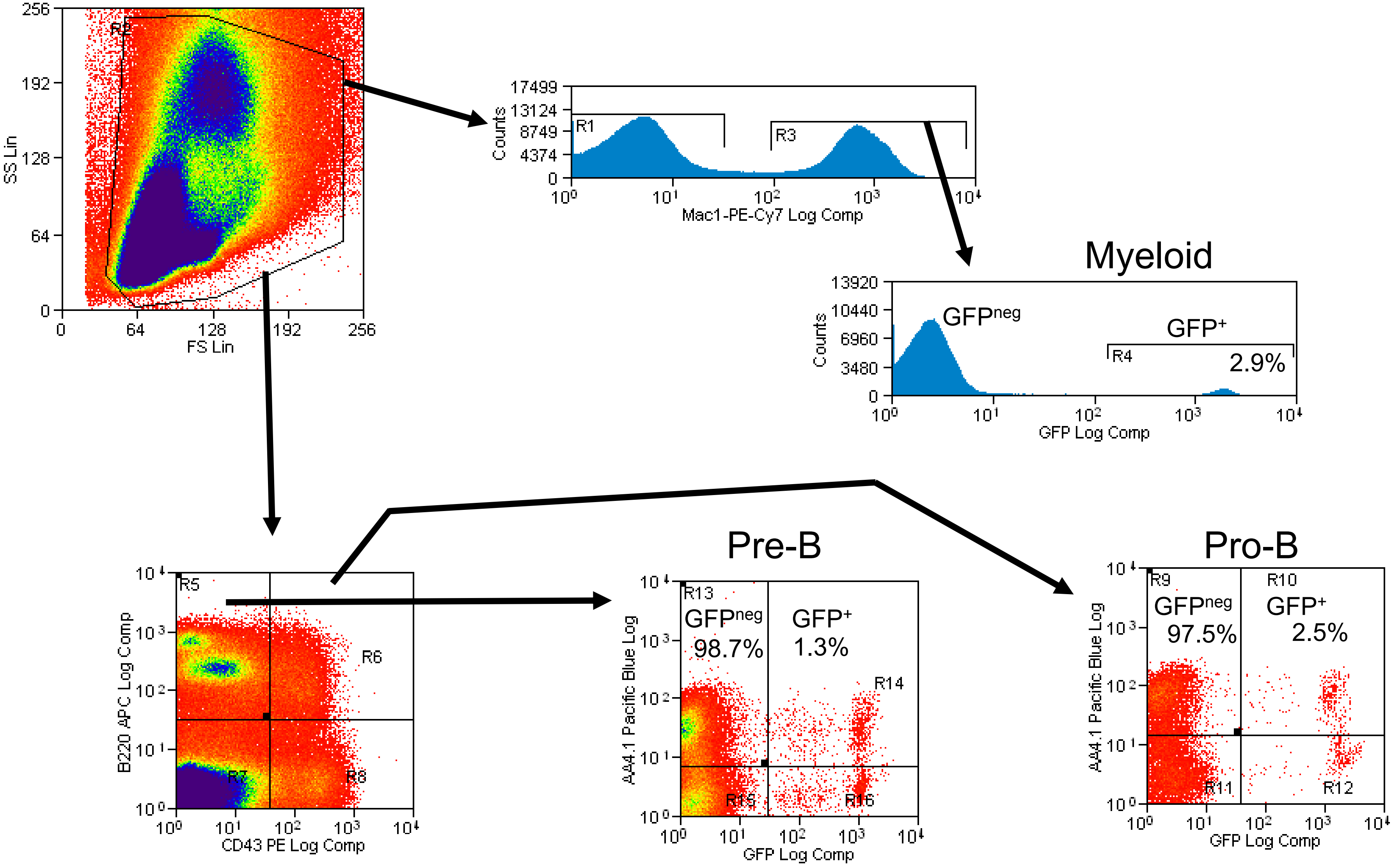

WT Irradiated

Figure S6B

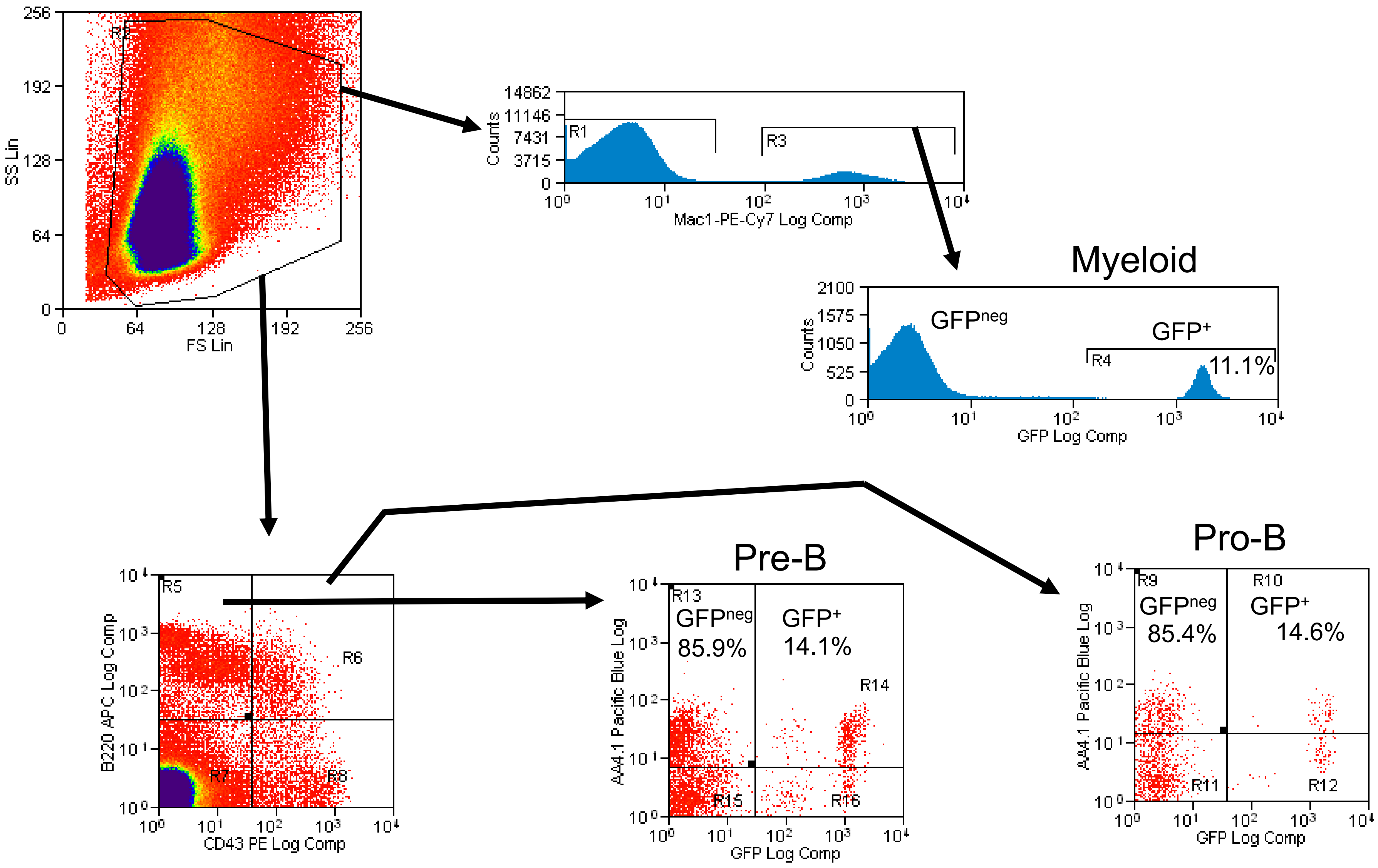

p53-/- mock

Figure S6C

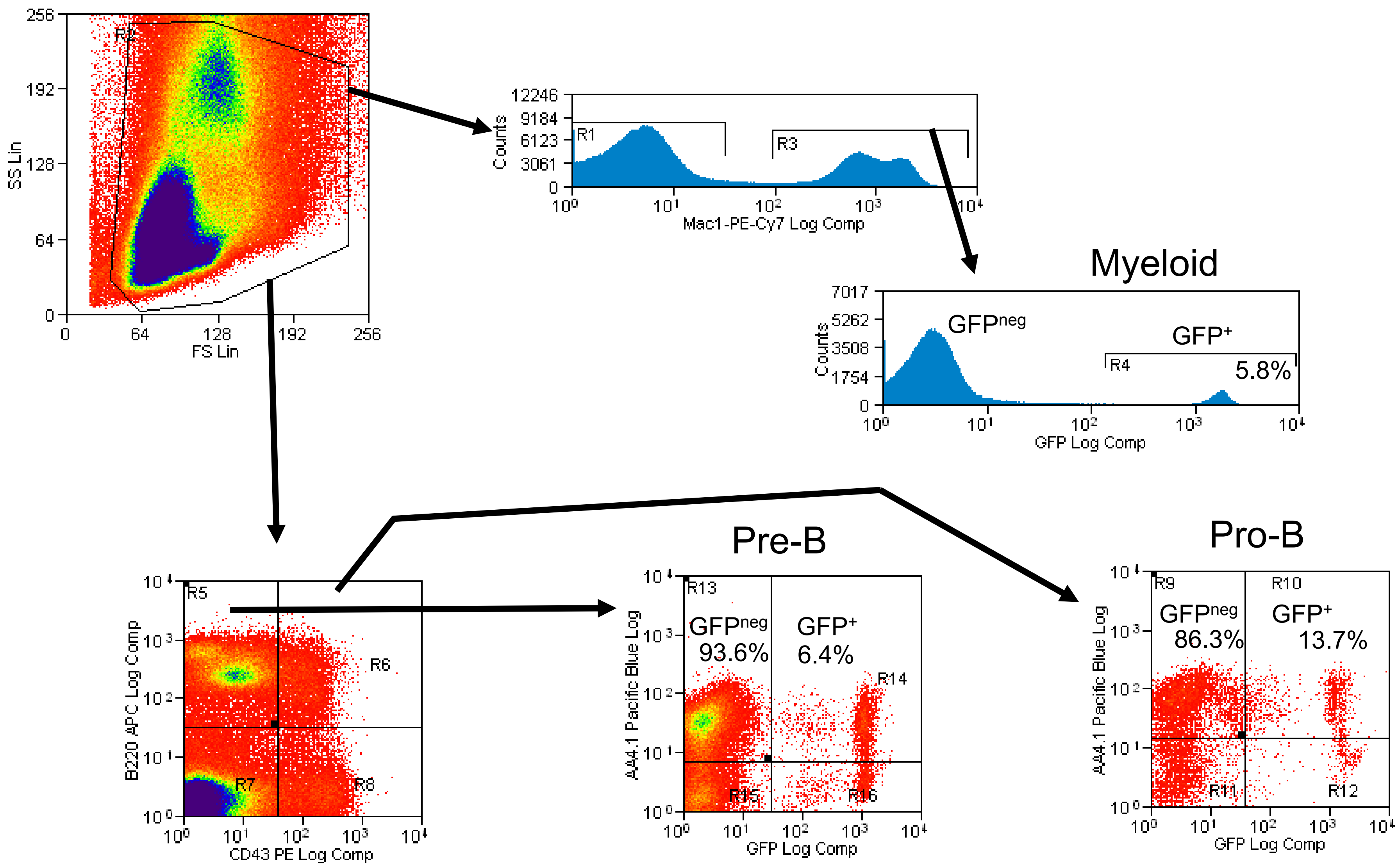

p53-/- Irradiated

Figure S6D

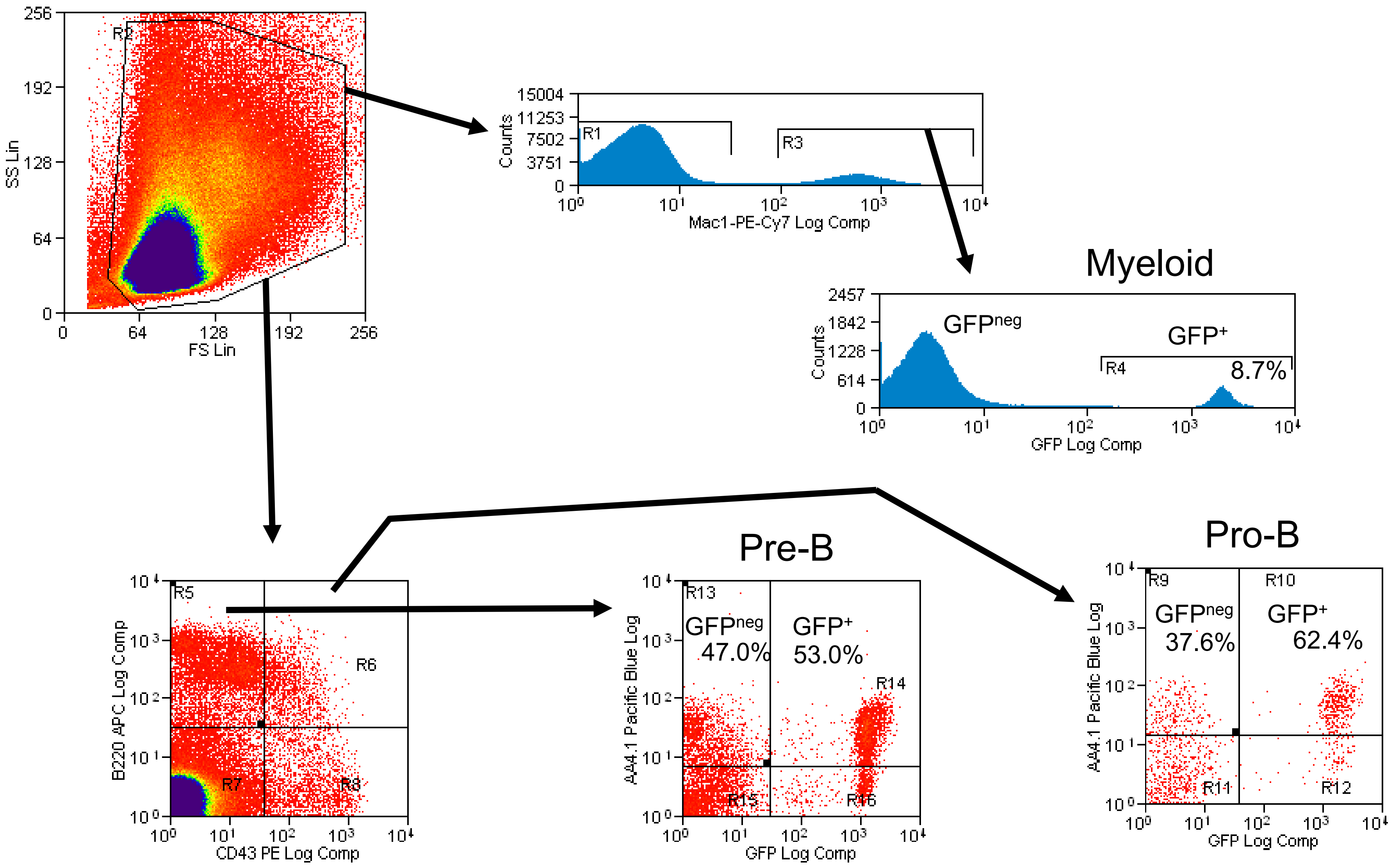

Figure S6E

p53-/- mock

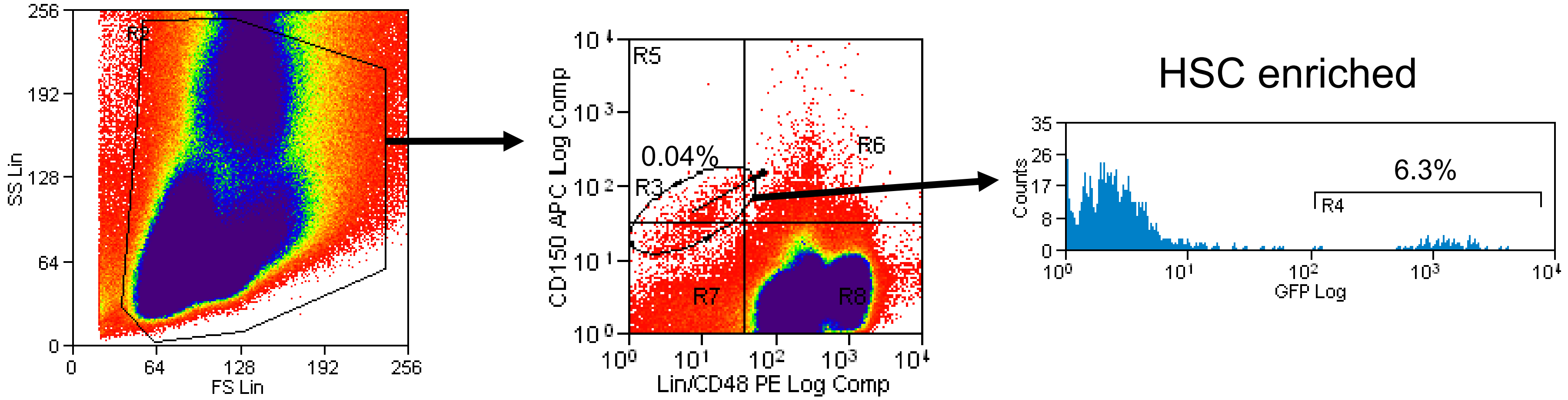

p53-/- Irradiated

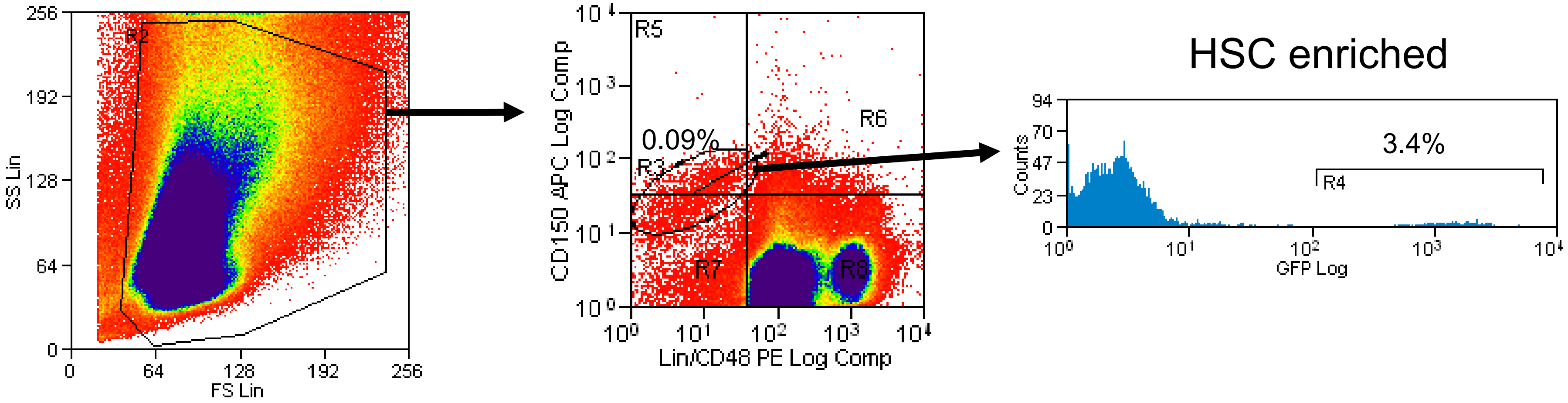

Supplement: Figure S6 — Examples of flow cytometric gating strategies for the analyses of pro-B, pre-B, myeloid, and HSC-enriched populations in the BM. Gating strategies are shown for the quantitation of GFP+ cells within the indicated populations used for Figures 3 and S5. (A) WT chimeric BM without irradiation (mock). (B) WT chimeric BM, 48 h post-irradiation. (C) p53−/− chimeric BM without irradiation. (D) p53−/− chimeric BM, 48 h post-irradiation. For (A–D), the percentages of GFP+ and GFPneg gates within the myeloid (Mac1+), pre-B (B220+CD93+CD43negMac1neg), and pro-B (B220+CD93+CD43+Mac1neg) cell compartments are indicated. (E) Examples of flow profiles and gating strategies for the LinnegCD48negCD150+ HSC-enriched population. The percentages of HSC-enriched cells (elliptical R3 gate, relative to total live cell gate) and of GFP+ cells within the HSC-enriched gate are indicated. Arrows indicate the gating strategy. (0.25 MB PDF) [file pbio.1000324.s006.pdf]
